# Catalytic innovation underlies independent recruitment of polyketide synthases in cocaine and hyoscyamine biosynthesis

**DOI:** 10.1038/s41467-022-32776-1

**Published:** 2022-08-25

**Authors:** Tian Tian, Yong-Jiang Wang, Jian-Ping Huang, Jie Li, Bingyan Xu, Yin Chen, Li Wang, Jing Yang, Yijun Yan, Sheng-Xiong Huang

**Affiliations:** 1 State Key Laboratory of Phytochemistry and Plant Resources in West China, and CAS Center for Excellence in Molecular Plant Sciences, Kunming Institute of Botany, Chinese Academy of Sciences, Kunming, 650201 China; 2 School of Chemistry and Chemical Engineering, Shaanxi Normal University, Xi’an, 710119 China; 3 University of Chinese Academy of Sciences, Beijing, 100049 China; 4 State Key Laboratory of Southwestern Chinese Medicine Resources, Innovative Institute of Chinese Medicine and Pharmacy, Chengdu University of Traditional Chinese Medicine, Chengdu, 611137 China

**Keywords:** Secondary metabolism, Biosynthesis, Biocatalysis

## Abstract

Tropane alkaloids such as hyoscyamine and cocaine are of importance in medicinal uses. Only recently has the hyoscyamine biosynthetic machinery become complete. However, the cocaine biosynthesis pathway remains only partially elucidated. Here we characterize polyketide synthases required for generating 3-oxo-glutaric acid from malonyl-CoA in cocaine biosynthetic route. Structural analysis shows that these two polyketide synthases adopt distinctly different active site architecture to catalyze the same reaction as pyrrolidine ketide synthase in hyoscyamine biosynthesis, revealing an unusual parallel/convergent evolution of biochemical function in homologous enzymes. Further phylogenetic analysis suggests lineage-specific acquisition of polyketide synthases required for tropane alkaloid biosynthesis in Erythroxylaceae and Solanaceae species, respectively. Overall, our work elucidates not only a key unknown step in cocaine biosynthesis pathway but also, more importantly, structural and biochemical basis for independent recruitment of polyketide synthases in tropane alkaloid biosynthesis, thus broadening the understanding of conservation and innovation of biosynthetic catalysts.

## Abstract (web summary)

Cocaine is a tropane alkaloid produced by *Erythroxylum novogranatense*. Here the authors identify two polyketide synthases involved in cocaine biosynthesis and provide insight into the parallel evolution of these enzymes in tropane alkaloids-producing plants.

## Introduction

**Fig. 1:**
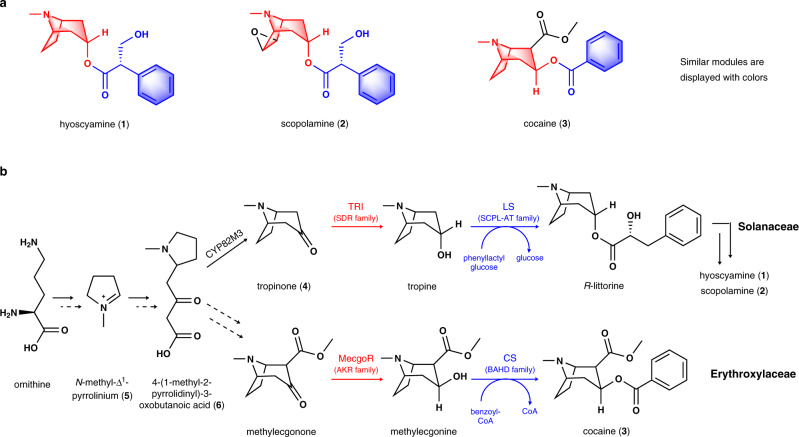
Chemical structures and partial biosynthesis steps of TAs. **a** Structural similarity among hyoscyamine, scopolamine and cocaine. **b** The enzymes of different families used for ketone reduction and subsequent esterification of tropane skeletons in Solanaceae and Erythroxylaceae. TRI tropinone reductase I, LS littorine synthase, MecgoR methylecgonone reductase, CS cocaine synthase, SDR short-chain dehydrogenase/reductase, SCPL-AT serine carboxypeptidase-like acyltransferase, AKR aldo-keto reductase, BAHD benzylalcohol *O*-acetyl transferase, anthocyanin *O*-hydroxycinnamoyl transferase, anthranilate *N*-hydroxycinnamoyl/benzoyl transferase, and diacetyl vindoline 4-*O*-acetyltransferase.

Tropane alkaloids (TAs) are a class of specialized metabolites that feature an 8-azabicyclo[3.2.1]octane ring. To date, more than 300 TAs have been identified from plant species in the Solanaceae, Convolvulaceae, Rhizophoraceae, Erythroxylaceae, and other families^1,2^, among which, hyoscyamine (**1**) and scopolamine (**2**) are lineage-specific compounds typical of Solanaceae while cocaine (**3**) is a signature metabolite of Erythroxylaceae (Fig. 1a). The plant extracts containing TAs were used as hallucinogens, poisons, and anesthetic in the Middle Ages in Europe and China^3^. Currently, hyoscyamine (**1**) and scopolamine (**2**) have a wide range of modern clinical applications, especially as anesthetic, antidote, and mydriatic, and atropine (racemic hyoscyamine) is considered one of the most efficacious, safe and cost-effective medicines for priority conditions by the World Health Organization^4^. Cocaine (**3**) (Goprelto and Numbrino) is recently approved by the FDA as a highly potent local anesthesia. In particular, tiotropium bromide (Spiriva), a derivative of scopolamine (**2**) used to treat lung diseases such as asthma, bronchitis, and emphysema, reached a record $2.136 billion in retail sales in 2020^5^.

As early as 1917, an initial presumption of TA biosynthesis was proposed by Robinson based on his landmark synthesis of tropinone (**4**)^6^. After a century of exploration, the complete biosynthetic route of **1** and **2** was recently fully resolved^7,8^, while their recombinant de novo biosynthesis was also described in yeast^7^. However, the cocaine (**3**) biosynthesis pathway was only partly elucidated (Fig. 1b)^2^. Comparisons between the established hyoscyamine (**1**) and cocaine (**3**) biosynthetic routes showed that Solanaceae and Erythroxylaceae plants use markedly different enzymes for similar biosynthetic reactions (Fig. 1b), although **1** and **3** share high chemical structure similarity (Fig. 1a). In Solanaceae, the biosynthesis of **1** and **2** employs tropinone reductases (TRs) of the short-chain dehydrogenase/reductase (SDR) enzyme family^9^ and littorine synthase (LS) of the serine carboxypeptidase-like acyltransferase (SCPL-AT) family^10^ for catalyzing reduction of the keto group and subsequent esterification (Fig. 1b). However, cocaine biosynthesis in Erythroxylaceae requires methylecgonone reductase (MecgoR) of the aldo-keto reductase (AKR) family^11^ and cocaine synthase (CS) of the BAHD acyltransferase (benzylalcohol *O*-acetyl transferase, anthocyanin *O*-hydroxycinnamoyl transferase, anthranilate *N*-hydroxycinnamoyl/benzoyl transferase, and diacetyl vindoline 4-*O*-acetyltransferase) family^12^ to catalyze the corresponding reduction and esterification steps (Fig. 1b). This substantial divergence raises several questions surrounding diversity and evolution of TA biosynthetic machineries in different plant lineages.

**Fig. 2:**
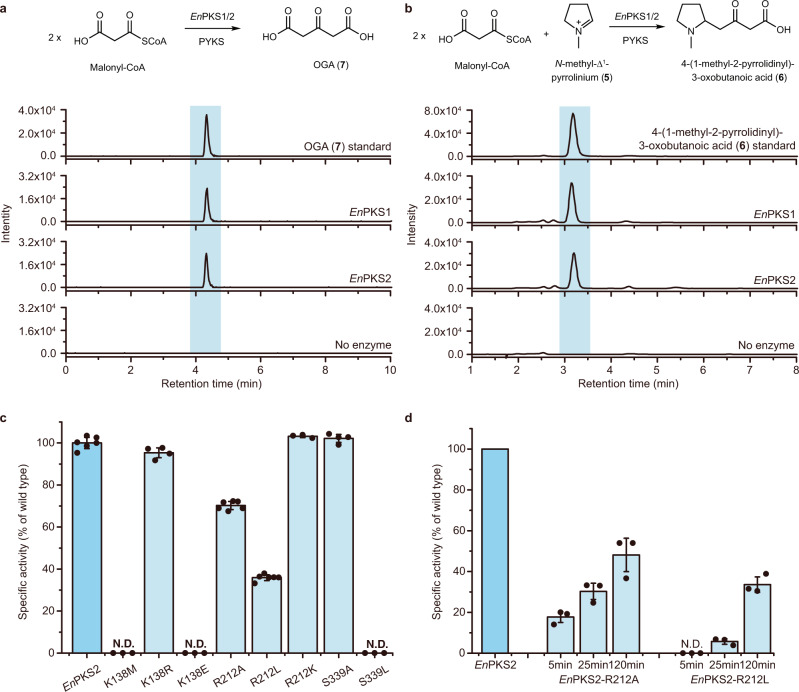
Identification of *En*PKSs. **a** LC-MS chromatograms at [M + Na]^+^ = 169.0107 of OGA (**7**) in enzymatic reactions using malonyl-CoA as substrate. A representative result of *n* = 3 independent experiments is shown. **b** LC-MS chromatograms at [M + H]^+^ = 186 of **6** in enzymatic reactions using malonyl-CoA and **5** as substrates. A representative result of *n* = 3 independent experiments is shown. **c** Production of OGA (**7**) catalyzed by wild type *En*PKS2 or its variants using 0.5 mM malonyl-CoA as substrate. Values are the means of the percent changes ± SD of *n* ≥ 3 independent experiments (dots; *n* = 6 for wild type *En*PKS2 and R212L; *n* = 3 for K138M, K138E, R212K, and S339L; *n* = 4 for K138R and S339A; *n* = 5 for R212A). **d** The catalytic activity of R212A and R212L mutants relative to the wild type *En*PKS2 at indicated time points using 1.0 mM malonyl-CoA as substrate. Values are the means of the percent changes ± SD of *n* = 3 independent experiments (dots).

Previous studies, based on isotopically labeled precursor feeding, have suggested that the condensation between *N*-methyl-Δ^1^-pyrrolinium (**5**) and malonyl-CoA gave rise to the crucial intermediate, 4-(1-methyl-2-pyrrolidinyl)-3-oxobutanoic acid (**6**) in both the hyoscyamine and cocaine biosynthetic pathways^13,14^ (Fig. 1b). Consistent with this possibility, three type III polyketide synthases (PKS), *Aa*PYKS1 (pyrrolidine ketide synthase) from *Anisodus acutangulus*, *Ab*PYKS1 from *Atropa belladonna*^15^, and *Ds*PYKS1 from *Datura stramonium* that all participate in **1** biosynthesis in solanaceous plants were identified^16^. Biochemical analysis showed that PYKSs use malonyl-CoA as the sole substrate to generate 3-oxo-glutaric acid (OGA, **7**) intermediate (Fig. 2a) which subsequently undergoes non-enzymatic Mannich condensation with *N*-methyl-Δ^1^-pyrrolinium (**5**) to produce racemic **6**^16^ (Fig. 1b). We thus sought to determine if a similar PKS also participated in the cocaine biosynthesis pathway in Erythroxylaceae plants.

In this work, by combining transcriptome annotations, gene expression pattern, and in vitro and in vivo enzyme activity assays, *En*PKS1 and *En*PKS2 responsible for cocaine biosynthesis in *Erythroxylum novogranatense* are identified. Structure-function analysis of *En*PKS1/2 deciphers a unique active site architecture distinct from that of *Aa*PYKS1 which catalyzes the same OGA-forming reaction in hyoscyamine biosynthesis. Phylogenetic tree analysis and active site residues exchange assay suggest that *En*PKS1/2 and *Aa*PYKS1 evolve independently in Solanaceae and Erythroxylaceae, two distant TA-producing plant lineages. Our work illustrates an unusual case of independent catalytic innovation, providing a fascinating arena for understanding the biochemical conservation and evolution in trajectories leading to chemotypic convergence in phylogenetically distant plants.

## Results

### Identification of *En*PKS1/2 in cocaine biosynthesis pathway

We identified seven candidates predicted *PKS* genes (*EnPKS1*-*7*) based on annotations in the transcriptomic sequencing data of *E. novogranatense*. Among them, *EnPKS1*-*3* exhibited the characteristic bud and young leaf-predominant expression pattern as that of two other enzyme genes (*MecgoR* and *CS*) well-established to function in cocaine biosynthesis (Supplementary Fig. 1). Meanwhile, *En*PKS3-5 showed the highest amino acid sequence identities (68–71%) with the OGA-forming PKS (*Aa*PYKS1) in hyoscyamine biosynthetic pathway (Supplementary Table 1). However, subsequent searches based on active site conservation were unsuccessful, since none of the seven *En*PKSs possessed the signature active site R134, which has been suggested to be critical for one-round malonyl-CoA condensation to produce OGA in *Aa*PYKS1^16^. We nevertheless succeeded in cloning these seven potential *PKS* genes from *E. novogranatense* cDNA library and expressed them in *Escherichia coli* (Supplementary Fig. 2) for further in vitro functional analysis. Notably, liquid chromatography-mass spectrometry (LC-MS) analysis showed two members of the seven *En*PKSs, *En*PKS1 and *En*PKS2, both showed catalytic activity in the formation of OGA (**7**) from malonyl-CoA (Fig. 2a and Supplementary Figs. 3 and 4a), the same as that of PYKSs in solanaceous plants. In the presence of *N*-methyl-Δ^1^-pyrrolinium (**5**), the resulting OGA (**7**) was further consumed to form 4-(1-methyl-2-pyrrolidinyl)-3-oxobutanoic acid (**6**) by condensation (Fig. 2b and Supplementary Fig. 4b). Moreover, by feeding deuterium-labeled intermediate [*N*-CD_3_] *N*-methyl-Δ^1^-pyrrolinium, transient expression of *En*PKS1, *En*PKS2 or *Aa*PYKS1 in combination with tropinone synthase *Ab*CYP82M3^15^ in tobacco (*Nicotiana benthamiana*) leaves resulted in formation of isotopically labeled tropinone (**4**), supporting in vivo role of *En*PKS1/2 in constructing tropane ring precursor (Supplementary Fig. 5).

Unexpectedly, amino acid sequence alignment showed that the conserved R134 residue in PYKSs was replaced by a threonine in both *En*PKS1 (T133) and *En*PKS2 (T133), which was interesting because the R134T variant of *Aa*PYKS1 showed significant decrease of OGA-forming activity in the previous study^16^. However, *K*cat/*K*m values for both *En*PKS1 and *En*PKS2 were shown as similar as for *Aa*PYKS1 (Supplementary Fig. 6 and Supplementary Table 2), suggesting that PKSs for TA biosynthesis in *E. novogranatense* may have evolved different residue(s) in the catalytic active site.

### Structural and biochemical basis for the OGA-forming activity in *En*PKS1/2

**Fig. 3:**
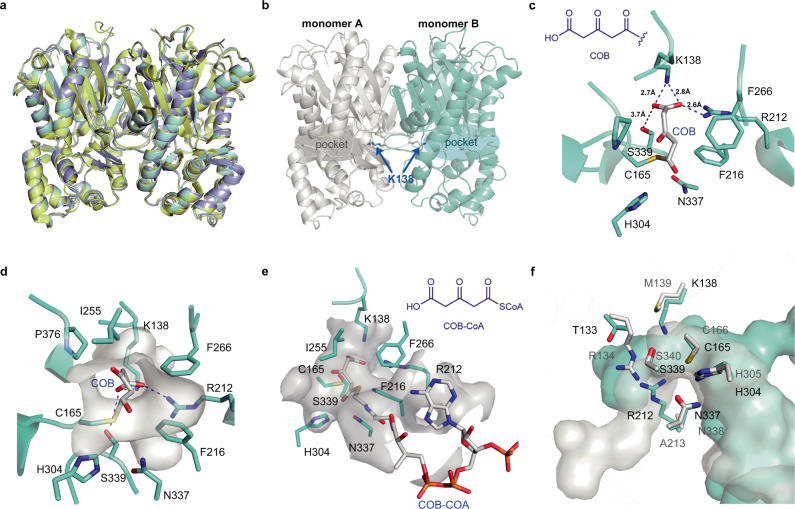
Crystal structures of *En*PKS1 and *En*PKS2. **a** Superposition of the *En*PKS1 (purple), *En*PKS2 (cyan), and *Aa*PYKS1 (yellow; PDB ID: 6J1M) structures. **b** Structure of *En*PKS2. K138 from another monomer participates in forming the active catalytic center. **c**, **d** Molecular docking of the COB intermediate with *En*PKS2. The *En*PKS2 catalytic pocket surface is colored in white. **e** Molecular docking of the COB-CoA intermediate in the *En*PKS2 active site. **f** Comparison of active site architecture between *En*PKS2 and *Aa*PYKS1. The catalytic pocket and key residues of *En*PKS2 and *Aa*PYKS1 in **f** are colored in cyan and white, respectively. N, O, S, and P atoms are colored blue, red, yellow, and orange, respectively. C atoms in *En*PKS2 residues and small molecules are colored as cyan and white, respectively.

To better understand the detailed catalytic mechanism of *En*PKS1 or *En*PKS2 that distinguish them from other solanaceous PYKSs, we determined the *En*PKS1 and *En*PKS2 crystal structures at 2.67Å and 2.62 Å resolution, respectively (*En*PKS1, PDB ID: 7F0G; *En*PKS2, PDB ID: 7F0E) (Supplementary Table 3). *En*PKS1 and *En*PKS2 showed homodimeric structures like other type III PKSs (Fig. 3a, b). Specifically, we observed a distinct catalytic pocket formed by R212, F216, I255 and S339 in monomer A, K138 in monomer B, and the type III PKS-conserved C165-H304-N337 triad (Fig. 3b–d and Supplementary Fig. 7). Since efforts to co-crystallize malonyl-CoA with *En*PKSs (*En*PKS1 and *En*PKS2) failed, we used molecular docking analysis with known *Aa*PYKS1 catalytic intermediates 4-carboxy-3-oxobutanoyl (COB) and 4-carboxy-3-oxobutanoyl-CoA (COB-CoA)^16^ in the predicted catalytic pocket of *En*PKS2 (Fig. 3c–e) to explore its potential catalytic mechanisms. As shown in Fig. 3d, F216 and I255 were predicted to govern the catalytic pocket size via hydrophobic interactions^17^. Importantly, K138 that protrudes from another monomer (Fig. 3b), in conjunction with R212 and S339 could form salt-bridge and hydrogen bonds with the carboxy group of COB/COB-CoA to fix the intermediates (Fig. 3c–e), a function previously suggested to be mediated by R134 and S340 in *Aa*PYKS1^16^.

To examine the role of these residues in the active site, site-directed mutants of *En*PKS2 were generated. We found that *En*PKS2 catalytic activity was abolished by methionine substitution at residue K138 (K138M) to mimic conventional cross-subunit interactions within type III PKS homodimers^17,18^, and the same result was also observed for K138E mutant (Fig. 2c and Supplementary Fig. 8a). However, the *En*PKS2 K138R mutant, in which arginine (R) could substitute functionally for lysine (K) in salt bridge formation, retained significant OGA-forming activity (Fig. 2c and Supplementary Fig. 8a). These results demonstrated the critical role of the salt-bridge interactions between K138 and the reaction intermediate in governing *En*PKS2 activity (Fig. 3c–e). Meanwhile, large-to-small (R212A) and polar-to-nonpolar (R212L) substitutions of R212 resulted in ~30% and ~64% decrease in *En*PKS2 activity, respectively, whereas functionally equivalent substitution (R212K) showed no difference (Fig. 2c and Supplementary Fig. 8b). In view of the decreased but still high catalytic activity remained in R212A and R212L mutants, further time-course study on the enzymatic reactions was conducted to obtain more quantitative information. As a result, it was found that the catalytic activity of R212A and R212L mutants relative to the wild type *En*PKS2 was closely associated with substrate (malonyl-CoA) concentration and incubation time of the enzymatic reaction (Supplementary Fig. 9). Consequently, before the wild type *En*PKS2 catalyzed reactions reached equilibrium, 61.5–82.2% and 94.2–96.6% decreases could be observed in R212A and R212L mutants, respectively (Fig. 2d and Supplementary Fig. 9). For S339, alanine substitution (S339A) did not lead to apparent decrease in *En*PKS2 activity (Fig. 2c and Supplementary Fig. 8c). These results indicated that K138 performs vital role in *En*PKS2 catalysis of OGA (**7**) formation and R212 serves as auxiliary residue influencing catalytic performance, while S339 is not essential for the catalytic process. Accordingly, K138 together with R212 likely fixed the COB/COB-CoA carboxy group via salt-bridge interactions efficiently, leaving the hydrogen bond interactions mediated by S339 unnecessary in controlling OGA-forming activity of *En*PKS2 (Fig. 3c). Additionally, a small-to-large substitution of S339 (S339L) caused the abolishment of *En*PKS2 activity (Fig. 2c and Supplementary Fig. 8c), possibly attributable to decreased space in the catalytic pocket (Fig. 3c–e).

### Plasticity of active sites recruited to control OGA-forming activity in *En*PKS1/2 and PYKS

Plant type III PKS is a superfamily sharing high similarity in amino acid sequence (30–95%) and overall protein structure^19^. Notably, it has been found that minor modulations of residues lining the catalytic pocket, where the conserved C-H-N catalytic triad is positioned, could result in dramatic changes in the pocket volume and shape, generating functionally different type III PKSs^19^. To gain further insight into the structural basis for controlling the specific one round of chain elongation in *En*PKS1/2, we compared their active-site architectures with type III PKSs pentaketide chromone synthase^20^ (PCS) and octaketide synthase^21^ (OKS) that catalyze four and seven rounds of chain elongation using malonyl-CoA as sole substrate, respectively (Supplementary Fig. 10). Notably, a narrow constriction defined by K138 and R212 was observed between the buried pocket and the active center (C165-H304-N337 triad) in *En*PKS1/2 (Supplementary Fig. 10). Together with the potential salt-bridge interactions between K138/R212 and the carboxy group of COB/COB-CoA mentioned above (Figs. 2c and 3), we proposed that K138 and R212 afford a narrowed catalytic tunnel and strong steric hindrance which prevent intermediate passage, thus terminating the reaction after one round of malonyl-CoA condensation.

**Fig. 4:**
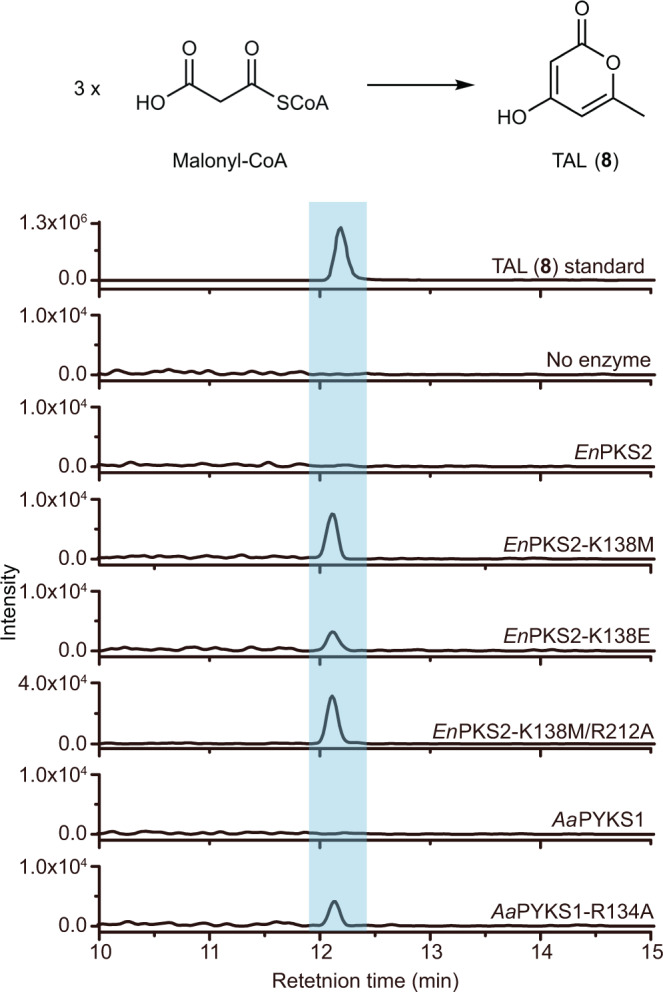
LC-MS chromatograms at [M + H]^+^ = 127.0390 ± 0.001 of TAL (**8**) in enzymatic reactions using malonyl-CoA as substrate. A representative result of *n* = 3 independent experiments is shown.

It has been revealed that a simple modulation of the active site residues could sterically alter the catalytic pocket, resulting in dramatic changes in polyketide chain length and product specificity^19^. Therefore, we generated different active site mutants of *En*PKS2 to examine whether they could synthesize longer polyketide products. It was intriguing to note that, concomitant with the loss of the OGA-forming activity, triacetic acid lactone (TAL, **8**, Supplementary Fig. 4c), the product expected from two rounds of malonyl-CoA extensions, was detected in the reactions catalyzed by *En*PKS2 mutants K138M, K138E, and K138M/R212A (Fig. 4 and Supplementary Fig. 11). Consistent with the suggested auxiliary role of R212 in *En*PKS2-catalyzed formation of OGA, the single mutation R212A or R212L did not result in production of TAL (Supplementary Fig. 11). Therefore, K138 is the key residue that controls polyketide chain elongation in *En*PKS2. In addition, when we replaced *Aa*PYKS1 R134^16^ with alanine (R134A), TAL (**8**) was also found in the corresponding catalytic reaction (Fig. 4 and Supplementary Fig. 11). By superposition, we found K138 of *En*PKS2 and R134 of *Aa*PYKS1 were located at non-equivalent positions in the structural scaffold (Fig. 3f). These findings suggested that different solutions have been developed in *En*PKS2 and *Aa*PYKS1 to achieve the steric constraints during polyketide chain elongation, resulting in the same OGA-forming activity. Moreover, except TAL (**8**), we did not detect any other longer polyketide products, most probably attributable to a still sterically restricted environment inside the catalytic pocket (Supplementary Fig. 10). Collectively, *En*PKS1 and *En*PKS2 in *E. novogranatense* adopt a distinct architecture by recruiting two specific amino acid residues, R212 from one monomer and K138 from another monomer to mediate the same catalytic activity as that of solanaceous PYKSs which employ R134 to limit malonyl-CoA elongation (Fig. 3f), indicating plasticity of active sites recruited to control OGA-forming activity in type III PKSs.

### Lineage-specific acquisition of PKSs required for TA biosynthesis

**Fig. 5:**
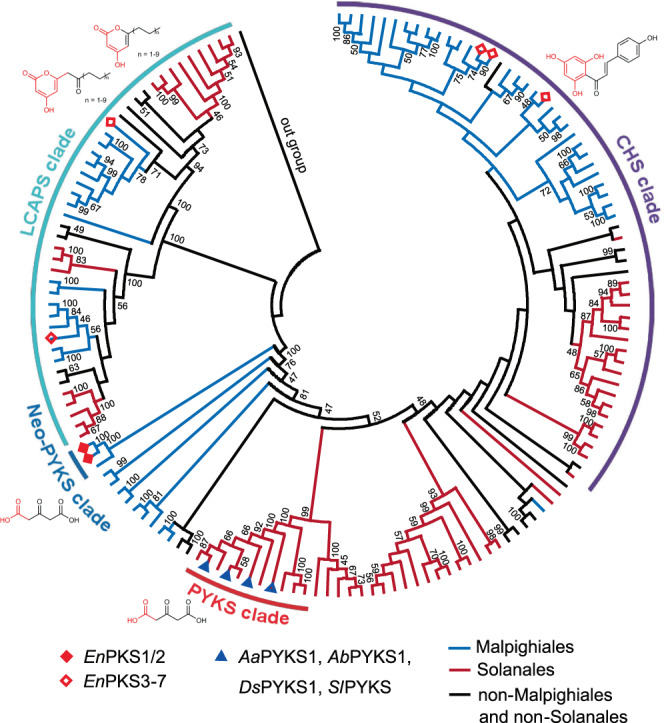
The phylogenetic tree of PKSs in Malpighiales and Solanales species. Chalcone synthase (CHS) clade; pyrrolidine ketide synthase (PYKS) clade in Solanales; neo-pyrrolidine ketide synthase (Neo-PYKS) clade in Malpighiales; long-chain alkyl *α*-pyrone synthase (LCAPS) clade. The structures of the PKS products are shown in the phylogenetic tree. A fully annotated phylogram is available in Supplementary Fig. 13.

The cocaine-producing plant family Erythroxylaceae is a clade in the Malpighiales, while hyoscyamine-producing Solanaceae is placed in the order Solanales. To further understand the evolutionary trajectories of PYKS and *En*PKS1/2 in Solanaceae and Erythroxylaceae plants, respectively, we performed phylogenetic analysis of genome-wide samples of PKSs from Malpighiales and Solanales species. Intriguingly, all putative TA-producing species of Solanales analyzed here (*Solanum tuberosum*, *Solanum lycopersicum*, *Capsicum annuum*, *Petunia inflata*, *Ipomoea triloba* and *Cuscuta campestris*) have PKSs that phylogenetically group with previously characterized PYKSs^16^ (*Aa*PYKS1, *Ab*PYKS1, *Ds*PYKS1, the PYKS clade) (Fig. 5 and Supplementary Figs. 12 and 13). Consistent with this finding, in vitro enzymatic assays indicated that the catalytic activity in OGA (**7**) production was also conserved in the corresponding *Sl*PYKS (Sly XP 004239898.1, *S. lycopersicum*^22^, tomato) (Supplementary Fig. 14), thus revealing a putative element required for TA (calystegines) biosynthesis in plants^23^. Notably, no *PYKS* gene was retained in tobacco genome^24^ (*Nicotiana attenuata*), which is consistent with the predominant production of nicotine alkaloid in this solanaceous plant rather than TAs (Fig. 5 and Supplementary Fig. 13). In Malpighiales, presumed ortholog of *En*PKS1/2 was only found in *Kandelia obovate*^25^ (*Ko*PKS, Kob GWHPACBH01626) (Fig. 5 and Supplementary Figs. 13 and 14), a putative TA-producing species (Rhizophoraceae)^26,27^ that is closely related to Erythroxylaceae plants. Moreover, sequence alignment on genome-wide samples of PKSs from Malpighiales and Solanales species revealed that K138 and R212, the active sites of *En*PKS1 and *En*PKS2, only appeared in *Ko*PKS (Supplementary Fig. 12), thus suggesting that *En*PKSs capable of OGA production in *E. novogranatense* emerged specifically in the last common ancestor of Erythroxylaceae and Rhizophoraceae plants in Malpighiales. We therefore named this clade of PKSs as Neo-PYKS since they have the same function as that of PYKSs in Solanales (Fig. 5).

There are different hypotheses regarding the origins of the variability in the functional residues that play the same role in homologous enzymes^28^. For *En*PKS2 and *Aa*PYKS1, they might evolve from the same ancestral PKS that recruited active sites independently in different phylogenetic lineages to acquire the same catalytic activity. Alternatively, parallel/convergent evolution of two ancestral PKSs with different function occurred. We then studied these two possibilities by switching the two different active site sets between *En*PKS2 and *Aa*PYKS1. Thus, two triple mutants, R134T/M139K/A213R of *Aa*PYKS1 and T133R/K138M/R212A of *En*PKS2, were generated. Interestingly, neither mutant showed the expected OGA-forming activity (Supplementary Fig. 15), raising the possibility that *En*PKS2 and *Aa*PYKS1 independently evolved from two primordial PKSs.

## Discussion

The biosynthetic route of cocaine has remained largely undetermined. In this study, we identified *En*PKSs responsible for tropane ring precursor construction in cocaine biosynthesis pathway, thus resolving a long-standing question as to whether the same set of enzymes are employed by the cocaine and hyoscyamine biosynthetic machineries. Previously, *Aa*PYKS1 was the unusual type III PKS identified in the hyoscyamine and scopolamine biosynthesis pathway that uses a specific catalytic pocket with the conserved R134 and S340 catalytic site to produce OGA (**7**)^16^. Although the divergence of enzymes responsible for tropane ring decoration has been characterized between Solanaceae and Erythroxylaceae plants^2^, we were still surprised that active site-based screening failed to identify PKSs involved in synthesis of the conserved tropane skeleton in *E. novogranatense*. Subsequent structure-function analysis revealed that spatially non-equivalent active sites were employed by *En*PKS1/2 (R212 and K138) and *Aa*PYKS1 (R134) to mediate the specific one-round chain elongation to generate OGA (**7**) (Fig. 3f and Supplementary Fig. 10), indicating independent catalytic innovation events may have occurred in PKS homologs.

Previous studies have widely addressed the participation of plant type III PKSs in the formation of a multitude of diverse scaffolds for medicinally valuable plant secondary metabolites, such as cannabinoids, curcuminoids, and quinolones^19,29,30^. In addition, parallel evolution in the PKS family that confers the same catalytic innovation in disparate plant lineages has been reported, such as the repeated independent emergence of stilbene synthases which arise from chalcone synthases^30^. Generally, the same or similar steric features of the active site can be observed in parallel-evolving homologs with identical catalytic properties^31,32^. The distinct differences in the active site cavities we found between *En*PKS1/2 and *Aa*PYKS1 illustrate a rare example of structural and functional evolution of PKS homologs, suggesting that independent mechanistic mutations have occurred in the ancestral PKSs to converge on the same OGA-forming activity. Recently, two PKSs (*Hs*PKS4 and *Pc*PKS1) with the conserved R134 and S340 residues as that of PYKSs, which produce OGA (**7**) for constructing the pelletierine block of Lycopodium alkaloids, were characterized in *Huperzia serrata* and *Phlegmariurus cryptomerianus*, respectively^33,34^ (Supplementary Fig. 12). Considering the potential versatility of OGA (**7**) in building diverse carbon skeletons via the non-enzymatic Mannich-like condensation^16,33,34^, we hypothesize that more PKSs will be identified in different secondary metabolite pathways based on the unique active sites (R212 and K138) in *En*PKSs. This unique example also alerts that the conventional conserved active sites-based search for enzymes catalyzing the same reaction could be ineffective in some cases, and that genome/transcriptome data combining with structural analysis will be the potent way to explore the diverse enzymatic elements behind the metabolism diversity in plants.

Phylogenetic analyses of the PKSs from both Malpighiales and Solanales species enabled a landscape view of the origins of OGA-producing PKSs in these two phylogenetically distant families. The distinct distribution of the Neo-PYKS clade and the PYKS clade in the phylogenetic tree (Fig. 5), the spatially non-equivalent active sites mentioned above, and especially, the non-interchangeable active sites between *En*PKS2 and *Aa*PYKS1, strongly suggested that *En*PKS1/2 (Neo-PYKS clade) and PYKSs (PYKS clade) originated independently from nonorthologous PKS progenitors. It means that the TA biosynthetic machineries in hyoscyamine-producing Solanaceae and cocaine-producing Erythroxylaceae probably emerged via independent recruitment of PKS homologs with divergent active site architecture that catalyze identical chemical reaction for tropane ring precursor construction. This unusual evolutionary solution to reach the same catalytic reaction underscores the expanding plasticity and adaptability of secondary metabolite catalysts. In addition, the discovery of unique PKSs in cocaine biosynthetic pathway and the insights into biochemical evolution of homologous enzymes can provide a theoretical framework for rational design of diverse protein scaffolds for synthetic biology and metabolic engineering in the innovation of specialized metabolites for medicinal and industrial applications.

## Methods

### Materials and experimental procedures

The reagents, solvents, and restriction enzymes were purchased from standard commercial sources and used directly. PCR amplifications were carried out on Bio-Rad T100 thermal cycler using Phanta® Super Fidelity DNA Polymerase (P505-d3, Vazyme, China). 3-oxo-glutaric acid (OGA, **7**, 165115-25g) and malonyl-CoA (M4263-5mg) were purchased from sigma-aldrich Co. (USA). Triacetic acid lactone (TAL, **8**) was purchased from Macklin (H811426-25g). Primer synthesis and DNA sequencing were performed by TsingKe Co. (China). LC-MS analysis was conducted on AGILENT 1290/6530 system and analyzed by Agilent MassHunter. HPLC analysis was conducted on a HITACHI Chromaster system equipped with a DAD detector, a YMC-Triart C_18_ column (I.D. 4.6 mm × 250 mm, Japan), and a flow rate of 1.0 mL/min at a column temperature of 25 °C. ChemBioDraw Ultra 14.0 was used for drawing chemical structures. OriginPro 9.0 was used for LC-MS and HPLC data visualization.

### Enzyme assay and kinetic parameters analysis

The standard assay mixture (100 μL) which contained potassium phosphate buffer (100 mM K_2_HPO_4_/KH_2_PO_4_, pH 8.0), 0.5 mM malonyl-CoA (for detection of formation of compound **6**, 1 mM **5** was also added) and 30 μg enzyme was incubated at 30 °C for 1 h. The reactions were stopped by adding 10 μL 20% HCl. After centrifugation, the supernatant was used for LC-MS or HPLC analysis. The analysis was performed using water with 0.1% formic acid as solvent A and methanol with 0.1% formic acid as solvent B. The injections were eluted with 5% B for 10 min.

According to measurements of initial reaction velocity, the reaction condition for enzyme kinetic assays was determined as follows: varied malonyl-CoA concentrations (20–1000 μM) and enzyme (the final concentration of enzyme was 1 ng/μL for *En*PKS1, 2 ng/μL for *En*PKS2 or 1 ng/μL for *Aa*PYKS1) in a final volume of 50 μL potassium phosphate buffer (100 mM K_2_HPO_4_/KH_2_PO_4_, pH 8.0) at 30 °C (the reaction time was 5 min for *En*PKS1, 8 min for *En*PKS2 or 5 min for *Aa*PYKS1). The reactions were quenched by adding 5 μL 20% HCl. Quantifications of the reaction products (OGA, **7**) were performed using HPLC. Kinetic parameter values were calculated by Graphpad Prism 7 software.

To identify products of PKS mutants, the reaction mixture contained 100 mM potassium buffer (K_2_HPO_4_/KH_2_PO_4_, pH 8.0), 1 mM malonyl-CoA and 25 μg enzyme in a final volume of 50 μL. After incubated at 30 °C for 90 min, the reactions were quenched by adding 5 μL 20% HCl. Enzyme reaction products analysis was performed on YMC-Triart C_18_ column (I.D. 4.6 mm × 250 mm), with a flow rate of 1 mL/min, using water with 0.1% formic acid as solvent A and acetonitrile as solvent B: 0–7 min 5% B; 7–12 min linear gradient from 5 to 100% B; 12–13 min 100% B; 13–15 min 5% B. The MS data were collected with positive ion mode (mass range: 50–1000 *m*/*z*).

### Crystallization and structure determination

Crystals of *En*PKS1 and *En*PKS2 were grown using sitting drop vapor diffusion method. Successful crystal growth could be observed in crystallization buffer (0.2 M calcium acetate, 20% w/v PEG3350, 0.1 M Tris-HCl pH 7.0) at 18 °C. Crystals diffraction data were collected from a single crystal at Shanghai Synchrotron Radiation Facility beamline 17U with a wavelength of 0.9795 Å at 100 K. The diffraction data were processed and scaled with XDS (BUILT = 20210205)^35^. The structures were solved by the molecular replacement method with structure of chalcone synthase (PDB: 6DXD). Initial model was build using Phenix 1.0^36^. Manual adjustment of the model was carried out using the program Coot-0.9.4^37^ and the models were refined by Phenix 1.0 and Refmac5^38^.

### Molecular docking of catalytic intermediates with *En*PKS2

Rigid molecular docking was performed in Autodock 4.2^39^. The ligands 4-carboxy-3-oxobutanoyl (COB) and 4-carboxy-3-oxobutanoyl-CoA (COB-CoA) were downloaded and extracted from the PDB database (PDB ID: 6J1M and 6J1N, respectively), and docked with the binding sites of *En*PKS2. The key parameters, such as grid number and algorithm, were set as default in docking, while the rotatable bond in the ligand was kept completely rigid. Finally, one hundred independent docking runs were generated and the complex structure with lower binding energy and favorable orientation was selected. PyMOL 2.4 (http://www.pymol.org) was used for viewing the molecular interaction and image processing.

### Reporting summary

Further information on research design is available in the Nature Research Reporting Summary linked to this article.

## Supplementary information

Supplementary Information

Reporting Summary

## Data Availability

The transcriptome datasets of *E. novogranatense* have been deposited in NCBI under accession numbers SRR15399168, SRR15399169, SRR15399170, SRR15399171, SRR15399172, SRR15399173, SRR15399174, SRR15399175, SRR15399176, SRR15399177, SRR15399178, SRR15399179, and SRR15399180. The gene sequences of *EnPKS1* and *EnPKS2* have been deposited in GenBank under accession numbers MZ819697 and MZ819696. The atomic models of *En*PKS1 and *En*PKS2 have been deposited in the Protein Data Bank under PDB IDs 7F0G and 7F0E. All data that support the findings of this study are available in the main text and the supplementary information. Source data are provided with this paper.

## Source data

Source Data

## Material availability

All unique materials are readily available from the corresponding author on request.
