# Supplementary material for: Catalytic innovation underlies independent recruitment of polyketide synthases in cocaine and hyoscyamine biosynthesis
Source: Nat Commun. 2022 Aug 25;13:4994. doi: 10.1038/s41467-022-32776-1 (PMC9411544; doi:10.1038/s41467-022-32776-1)
Supplement: Supplementary file 1 — Supplementary Information [file 41467_2022_32776_MOESM1_ESM.pdf]

Supplementary Information

Catalytic innovation underlies independent recruitment of polyketide synthases in cocaine and hyoscyamine biosynthesis

Tian Tian, Yong-Jiang Wang, Jian-Ping Huang, Jie Li, Bingyan Xu, Yin Chen, Li Wang, Jing Yang, Yijun Yan, Sheng-Xiong Huang

Correspondence to: sxhuang@mail.kib.ac.cn

This PDF file includes:

Supplementary Methods

Supplementary Figures 1 to 19

Supplementary Tables 1 to 4

Supplementary References

Supplementary Methods

Transcriptome sequencing

Six tissues (bud, young leaf, old leaf, stem, flower and root) of *E. novogranatense* were used for RNA sequencing. The RNA Illumina sequencing libraries were generated using NEBNext Ultra^{TM} RNA Library Prep Kit (NEB, USA). The corresponding cDNA libraries were then sequenced on Illumina NovaSeq 6000 platform.

Gene cloning and protein expression

Seven *EnPKSs* were amplified from cDNA of *E. novogranatense* using primer pairs listed in Supplementary Table 4. The codon-optimized *SlPYKS* gene of *Solanum lycopersicum* (tomato) was synthesized by GeneWiz (Suzhou, China). These *PKS* genes were cloned into pET28a expression vector, and then transformed into *E. coli* DH5*α*. After selected by LB agar plates supplemented with kanamycin (50 μg mL^{-1}), constructs were confirmed by sequencing. Protein expressions were performed using *E. coli* Rosetta (DE3). These *E. coli* strains were cultured in LB liquid medium supplemented with chloramphenicol (25 μg mL^{-1}) and kanamycin (50 μg mL^{-1}) at 37 °C with shaking at 200 rpm to an OD_{600} value of 0.6 - 0.8. Then target protein was induced with 0.5 mM IPTG followed by subsequent growth at 16 °C for 18 - 20 h. The cell pellets were collected and resuspended in buffer A (20 mM imidazole, 50 mM Tris, 300 mM NaCl, 10% glycerol, pH 8.0). The resulting suspensions were lysed by sonication. After centrifugation at 68,905 x g for 30 min, the supernatants were loaded on the HisTrap FF 5 mL column. The targeted protein fractions were obtained by a gradient elution of buffer A and buffer B (500 mM imidazole, 50 mM Tris, 300 mM NaCl, 10% glycerol, pH 8.0) and then concentrated by Amicon Ultra-4 centrifugal filters (Ultracel, 10,000 NMWL). The targeted proteins were finally dissolved in storage buffer (100 mM NaH_2PO_4, 10% glycerol, pH 7.0).

Site mutation of *En*PKS2

The wild pET28a-*EnPKS2* plasmid was used as template to construct site-mutated expression plasmid with primers listed in Supplementary Table 4. Due to poor solubility of site-mutated *En*PKS2 proteins in pET28a expression system, the expression plasmid was replaced by pCold-TF plasmid which contain a molecular chaperone trigger factor to promote the production of soluble site-mutated *En*PKS2 proteins. The reaction mixture for the enzymatic assay containing potassium phosphate buffer (50 mM K_2HPO_4/KH_2PO_4, pH 8.0), 0.5 mM malonyl-CoA and 140 μg enzyme in a 100 μL reaction volume at incubated at 30 °C for 45 min. The reactions were stopped by adding 10 μL 20% HCl.

The time course assays of wild type *En*PKS2, variants R212A and R212L were monitored by HPLC at 242nm. Enzymatic reactions were carried out in 100 mM potassium buffer (pH 8.0) with different final concentrations of malonyl-CoA (0.5 and 1.0 mM) in a final volume of 50 μL and incubated at 30 °C for various times (5 min, 10 min, 25 min, 40 min, 60 min, 90 min and 120 min). The reactions were initiated by addition of 20 μg enzyme, and were stopped by adding 5 μL 20% HCl. The specific activities of R212A and R212L mutants (61.5%-82.2% decreases for R212A and 94.2%-96.6% decreases for R212L) were counted before the reactions reached equilibrium.

Synthesis of *N*-methyl-Δ^1-pyrrolinium (5)

The *N*-methyl-Δ^1-pyrrolinium was synthesized, purified and stocked in our lab [1]. The [*N*-CD_3] *N*-methyl-Δ^1-pyrrolinium was synthesized in a similar manner with minor modifications. Sodium hydride (60% dispersion in oil, 122 mg, 3.05 mmol) was added to a solution of **s-1** (666 mg, 2.55

mmol) in DMF (8 mL). After 1 h, [CD_3]-iodomethane (0.19 mL, 3.06 mmol) was added and the mixture stirred at rt for 18 h. The reaction mixture was quenched with water (50 mL) and extracted with EtOAc (3 × 25 mL). The organic layer was washed with brine (saturated NaCl solution, 6 × 30 mL), dried with Na_2SO_4, and evaporated in vacuo. The pure product was obtained by column chromatography on silica gel eluting with petroleum ether/EtOAc (5:1) to afford compound **s-2** (0.52 g, 73%). ^{1}H NMR (400 MHz, $CDCl_3$): δ 4.49 (s, 1 H), 3.68–3.60 (m, 2 H), 3.53–3.45 (m, 2 H), 3.22 (brs, 2 H), 1.62–1.58 (m, 4 H), 1.45 (s, 9 H), 1.20 (t, J = 7.0 Hz, 6 H); ^{13}C NMR (100 MHz, $CDCl_3$): δ 155.8, 102.6, 79.1, 61.1, 30.8, 28.4, 23.2, 15.3. Compound **s-2**: HRMS (*m/z*): [M + Na]$^+$ calcd. for $C_{14}H_{26}D_3NO_4Na^+$, 301.2177; found, 301.2174 (Supplementary Figs. 16-18).

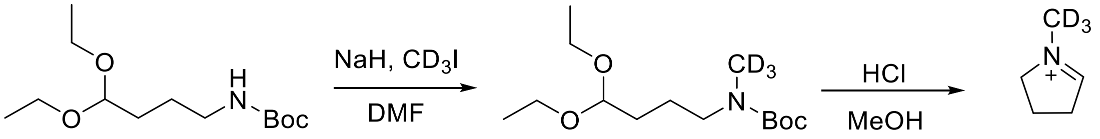

s-1 **s-2** [*N*-CD_3] *N*-methyl-Δ^1-pyrrolinium

The solution of compound **s-2** (50 mg, 0.18 mmol) in MeOH (300 μL) was cooled in an ice bath for 10 min. Then, a solution of HCl (4 M, 300 μL) was added dropwise. The mixture was stirred at 0 °C for 20 min and then at rt for 16 h to afford [*N*-CD_3] *N*-methyl-Δ^1-pyrrolinium. HRMS (*m/z*): [M]$^+$ calcd. for $C_5H_7D_3N^+$, 87.0996, found, 87.0998 (Supplementary Fig. 19).

Transient expression of *En*PKS1/2 in *N. benthamiana*

EnPKS1, *EnPKS2*, *AaPYKS1*, *AbCYP82M3* (GenBank accession number: MH292964.1) and *AtATR1* (Plant P450 redox partner gene, GenBank accession number: NM_118585.4) were constructed into the pEAQ-HT vector. The resulting plasmids were then transformed into *Agrobacterium tumafaciens* (LBA4404) using the freeze-thaw method. Transformants were grown on LB plates supplemented with rifampicin (20 μg mL^{-1}), streptomycin (50 μg mL^{-1}) and kanamycin (50 μg mL^{-1}) at 30 °C. The cells were suspended in MMA buffer (10 mM MES, pH = 5.6, 10 mM $MgCl_2$, 150 μM acetosyringone) to obtain *Agrobacterium* suspensions (OD_{600} = 0.3 for each strain with different gene). The *Agrobacterium* suspensions were incubated at room temperature for 40 min, and then infiltrated into *N. benthamiana* leaves. After 4 days, the substrate (1 mM [*N*-CD_3] *N*-methyl-Δ^1-pyrrolinium solution, pH = 6.0) was also infiltrated into previously *Agrobacterium*-infiltrated leaves. [*N*-CD_3] *N*-methyl-Δ^1-pyrrolinium was used to avoid the interference of endogenous *N*-methyl-Δ^1-pyrrolinium produced by *N. benthamiana*. Leaves were harvested 1 day later and were then grinded in liquid nitrogen. The powders were extracted with methanol. The organic layers were evaporated in vacuo and were dissolved in acid aqueous solution (pH = 2.0). The aqueous layers were extracted with chloroform. Then, the pH of resulting aqueous layers were adjusted to 9.0 with 1 M NaOH and then extracted with chloroform. The organic layers were evaporated in vacuo and were dissolved in methanol. The methanol solutions were used for LC-MS analysis. The analysis was performed on a YMC-Triart C_{18} column (I.D. 4.6 mm × 250 mm), using water with 0.1% formic acid as solvent A and acetonitrile with 0.1% formic acid as solvent B. The injections were eluted with 10% B for 10 min, with a flow rate of 1 mL/min. The MS data were collected with positive ion mode (mass range: 50 - 400 *m/z*).

The construction of phylogenetic tree

Phylogenetic tree was constructed by MEGA (version 6) with neighbor-joining method (parameters: 1000 bootstrap replications, *p*-distance substitution model and partial deletion gaps

data treatment with 90% site coverage cutoff). *Arabidopsis thaliana At*1G01120.1, which is annotated as 3-ketoacyl-CoA synthase [2], was chosen as outgroup. GenBank accession numbers of *Ds*PYKS1, *Ab*PYKS1 and *Ms*CHS are QEP99906.1, AYU65302.1 and P30074.1, respectively. Four PKSs from *A. acutangulus* and seven PKSs from *E. novogranatense* were obtained from their respective transcriptome data. 138 PKSs were chosen by Blastp search of protein sequences from the genomes of seven species (*Kandelia obovate* (https://ngdc.cncb.ac.cn/gwh/Assembly/990/show), *Populus trichocarpa* (https://www.ncbi.nlm.nih.gov/genome/?term=Populus+trichocarpa), *Hevea brasiliensis* (https://www.ncbi.nlm.nih.gov/genome/?term=Hevea+brasiliensis), *Ricinus communis* (https://www.ncbi.nlm.nih.gov/genome/?term=Ricinus+communis), *Salix brachista* (https://www.ncbi.nlm.nih.gov/genome/?term=Salix+brachista), *Linum usitatissimum* (https://phytozome-next.jgi.doe.gov/info/Lusitatissimum_v1_0) and *Manihot esculenta* (https://www.ncbi.nlm.nih.gov/genome/441?genome_assembly_id=1703475)) in Malpighiales, seven species (*Solanum lycopersicum* (https://phytozome-next.jgi.doe.gov/info/Slycopersicum_ITAG3_2), *Nicotiana attenuata* (https://www.ncbi.nlm.nih.gov/genome/13243), *Capsicum annuum* (https://db.cngb.org/search/project/CNPhis0000547/), *Petunia inflata* (https://solgenomics.net/organism/Petunia_inflata/genome), *Solanum tuberosum* (https://solgenomics.net/ftp/potato_genome/annotation/ITAG1.0/), *Cuscuta campestris* (http://plabipd.de/portal/cuscuta-campestris) and *Ipomoea triloba* (https://datadryad.org/stash/dataset/doi:10.5061/dryad.b9m61cg)) in Solanales, and other four species (*A. thaliana* (https://phytozome-next.jgi.doe.gov/info/Athaliana_Araport11), *Catharanthus roseus* (https://datadryad.org/stash/dataset/doi:10.5061%2Fdryad.08vv50n), *Gelsemium sempervirens* (https://datadryad.org/stash/dataset/doi:10.5061/dryad.08vv50n) and *Tectona grandis* (https://datadryad.org/stash/dataset/doi:10.5061/dryad.77b2422)).

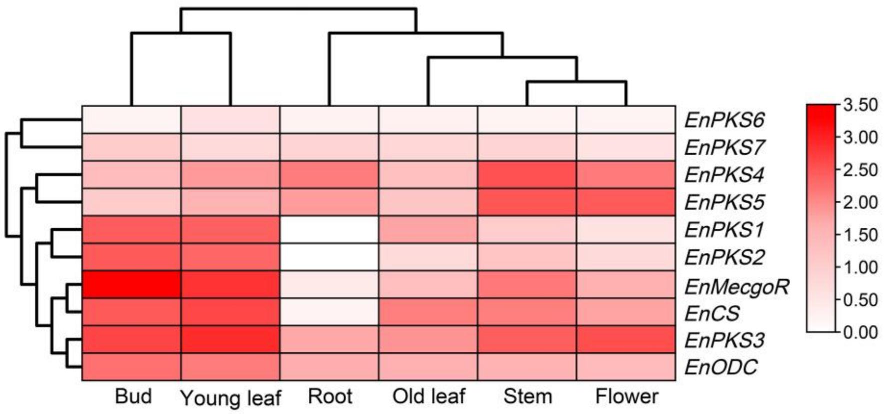

Supplementary Figure 1. Expression patterns of seven *PKS* genes and functional characterized genes (*EnMecgoR* and *EnCS*) involved in cocaine biosynthesis in different tissues of *E. novogranatense*. *EnPKS1* and *EnPKS2* showed the characteristic bud and young leaf-predominant expression pattern as *EnMecgoR* and *EnCS*.

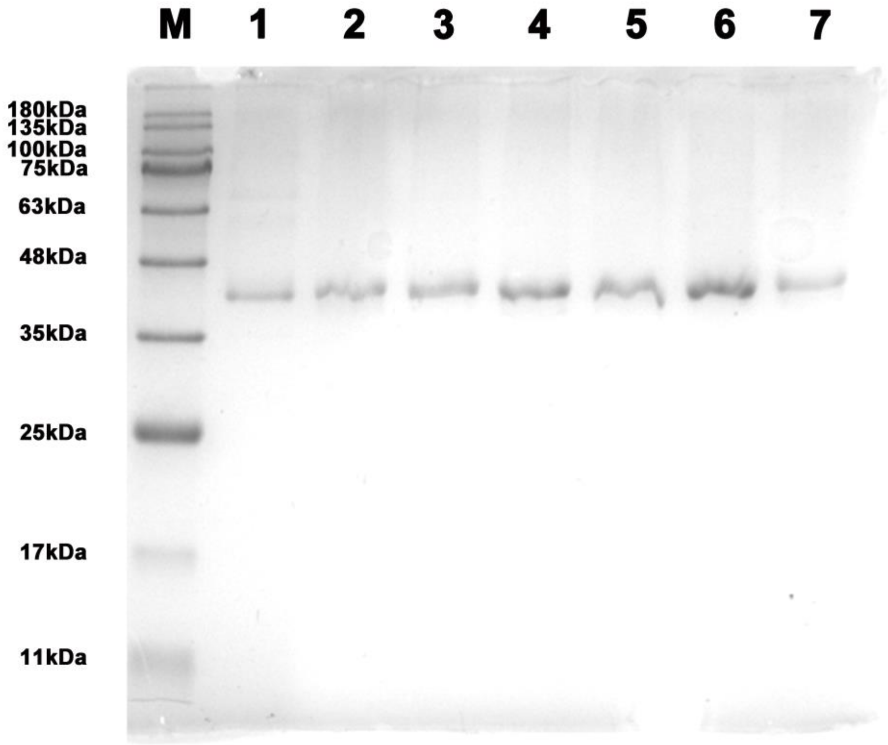

Supplementary Figure 2. The electrophoresis of purified proteins. M: protein molecular marker; 1: *En*PKS1; 2: *En*PKS2; 3: *En*PKS3; 4: *En*PKS4; 5: *En*PKS5; 6: *En*PKS6; 7: *En*PKS7. The figure is the representative of results from n = 3 independent experiments.

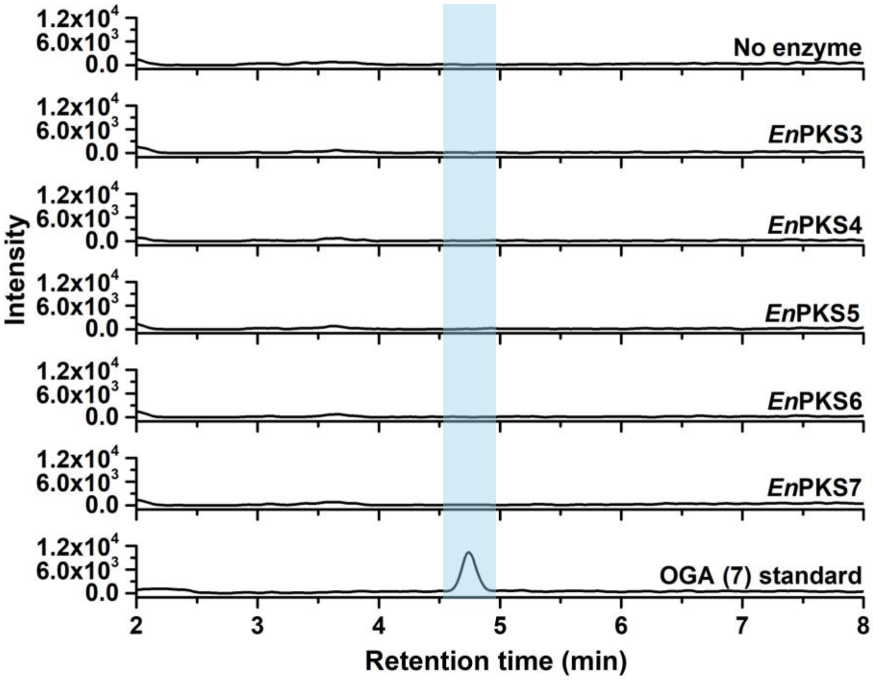

Supplementary Figure 3. LC-MS chromatograms at $[M + Na]^+ = 169$ of OGA (7) in enzymatic reactions using malonyl-CoA as substrate. A representative result of *n* = 3 independent experiments is shown.

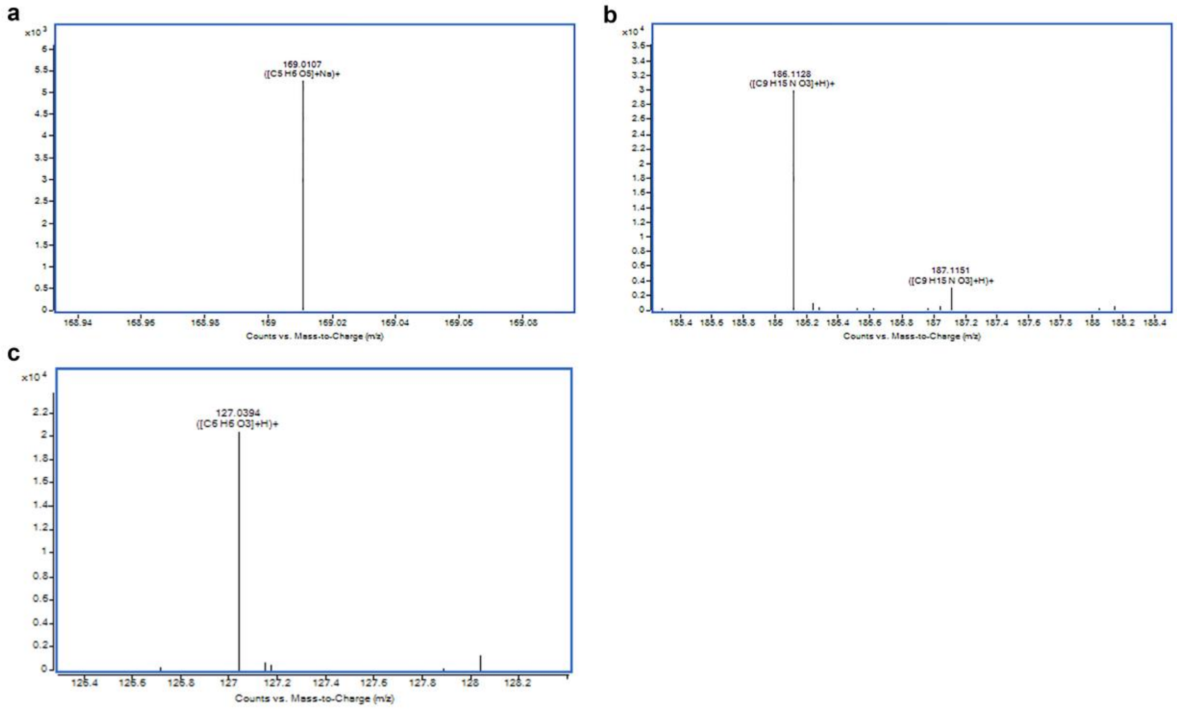

Supplementary Figure 4. HRMS analysis of OGA (7) and 4-(1-methyl-2-pyrrolidinyl)-3-oxobutanoic acid (6). (**a**) The HRMS of OGA (**7**), (*m/z*): $[M + Na]^+$ calcd. for $C_5H_6O_5Na^+$, 169.0107; found, 169.0107. (**b**) The HRMS of 4-(1-methyl-2-pyrrolidinyl)-3-oxobutanoic acid (**6**), (*m/z*): $[M + H]^+$ calcd. for $C_9H_{16}NO_3^+$, 186.1125; found, 186.1128. (**c**) The HRMS of TAL (**8**), (*m/z*): $[M + H]^+$ calcd. for $C_6H_7O_3^+$, 127.0390; found, 127.0394.

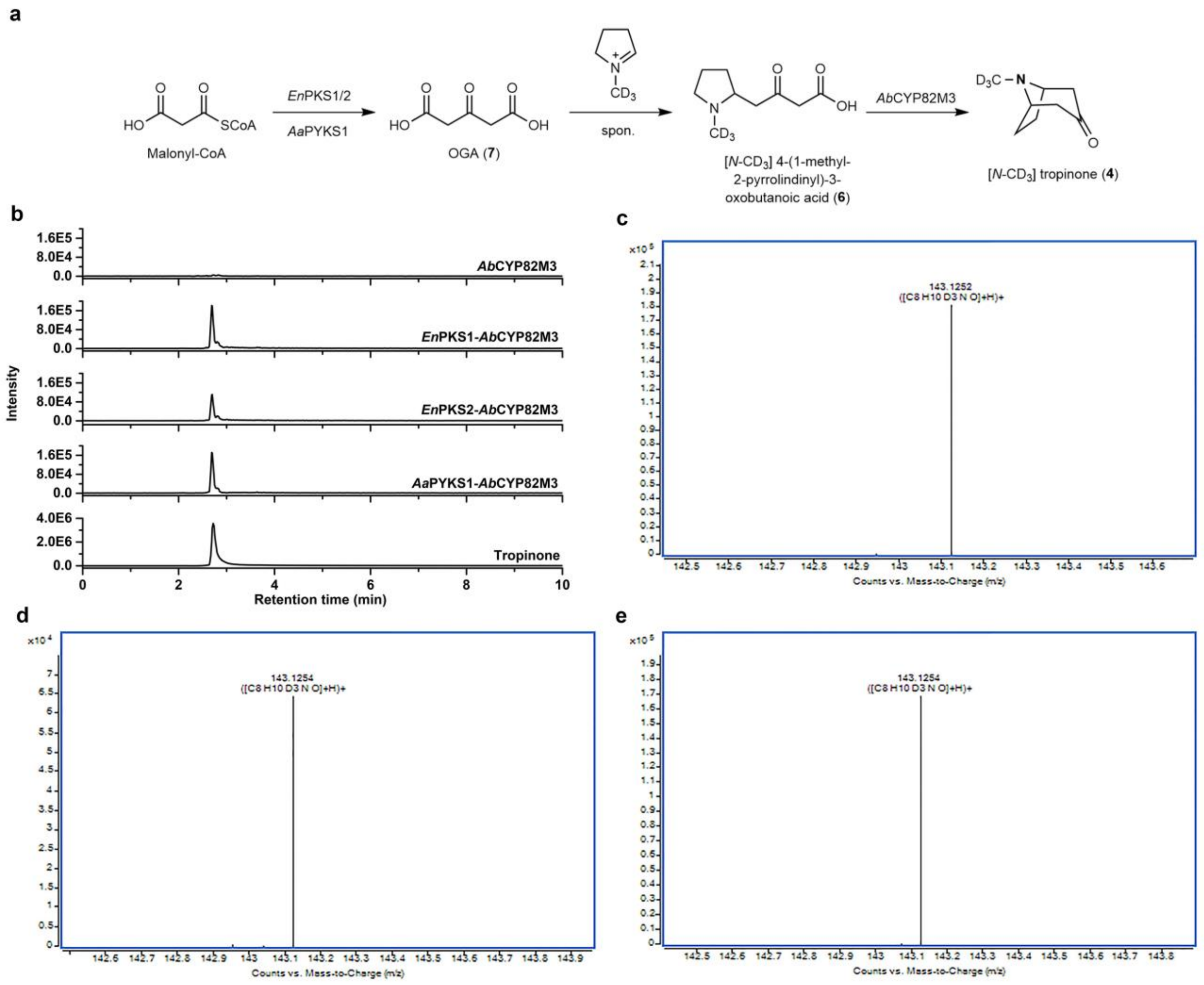

Supplementary Figure 5. The functional analysis of *En*PKS1, *En*PKS2 or *Aa*PYKS1 in vivo. (**a**) The formation process of [*N*-CD_3] tropinone from malonyl-CoA and [*N*-CD_3] *N*-methyl-Δ^1-pyrrolinium catalyzed by *En*PKS1/2 (*Aa*PYKS1) and *Ab*CYP82M3. (**b**) The LC-MS analysis of tropinone produced by *En*PKS1, *En*PKS2 or *Aa*PYKS1 combined with *Ab*CYP82M3 in tobacco *(N. benthamiana)*. The products for negative control (*Ab*CYP82M3) and PKS assays (*Ab*CYP82M3 in combination with *En*PKS1, *En*PKS2, or *Aa*PYKS1) were detected at *m/z* =143.1258 ± 0.0010 for [*N*-CD_3] tropinone; The tropinone was detected at *m/z* =140.1070 ± 0.0010. A representative result of n = 2 independent experiments is shown. (**c**) The HRMS of [*N*-CD_3] tropinone obtained by the assay of *En*PKS1 and *Ab*CYP82M3, (*m/z*): $[M + H]^+$ calcd. for $C_8H_{11}D_3NO^+$, 143.1258; found, 143.1252. (**d**) The HRMS of [*N*-CD_3] tropinone obtained by the assay of *En*PKS2 and *Ab*CYP82M3, (*m/z*): $[M + H]^+$ calcd. for $C_8H_{11}D_3NO^+$, 143.1258; found, 143.1254. (**e**) The HRMS of [*N*-CD_3] tropinone obtained by the assay of *Aa*PYKS1 and *Ab*CYP82M3, (*m/z*): $[M + H]^+$ calcd. For $C_8H_{11}D_3NO^+$, 143.1258; found, 143.1254.

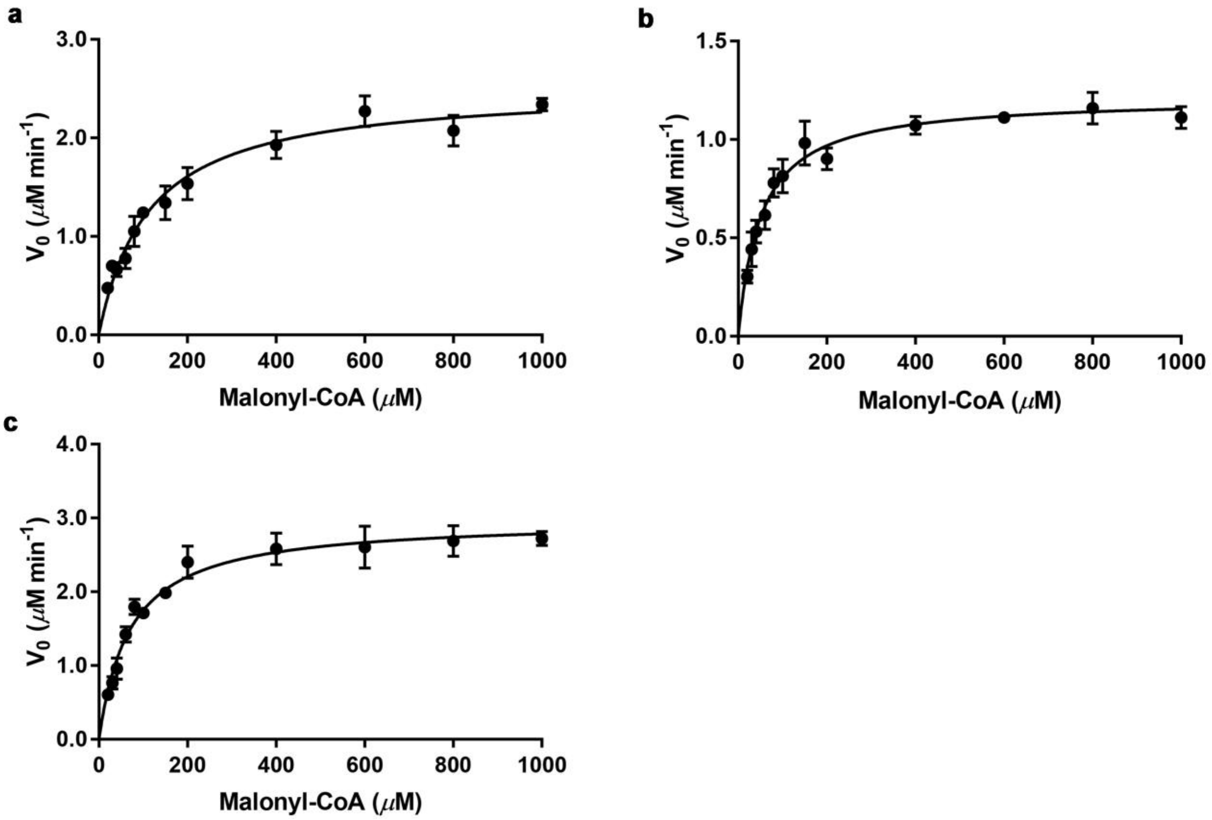

Supplementary Figure 6. Michaelis-Menten kinetic plots of PKSs towards malonyl-CoA. (**a**) *En*PKS1; (**b**) *En*PKS2; (**c**) *Aa*PYKS1. The abscissa represents the final concentration of malonyl-CoA in the reaction; The ordinate represents the production velocity of OGA (**7**). The plots were obtained by nonlinear regression curve-fit according to the Michaelis-Menten model in GraphPad Prism 7 software. Values are the means ± SD of n = 3 independent experiments, except for 100 µM malonyl-CoA in **a** (n = 2), 60 µM and 200 µM malonyl-CoA in **b** (n = 2), 30 µM, 40 µM, 80 µM, 100 µM and 150 µM malonyl-CoA in **b** (n = 4), and 30 µM, 40 µM and 60 µM malonyl-CoA in **c** (n = 4).

.

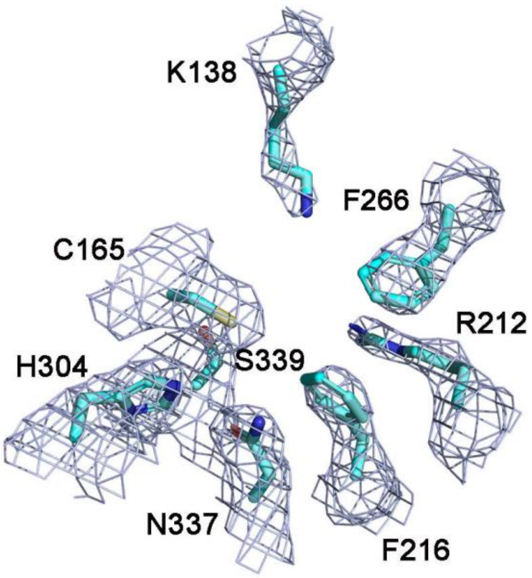

Supplementary Figure 7. The electron density map of *En*PKS2 in the area of the active site.

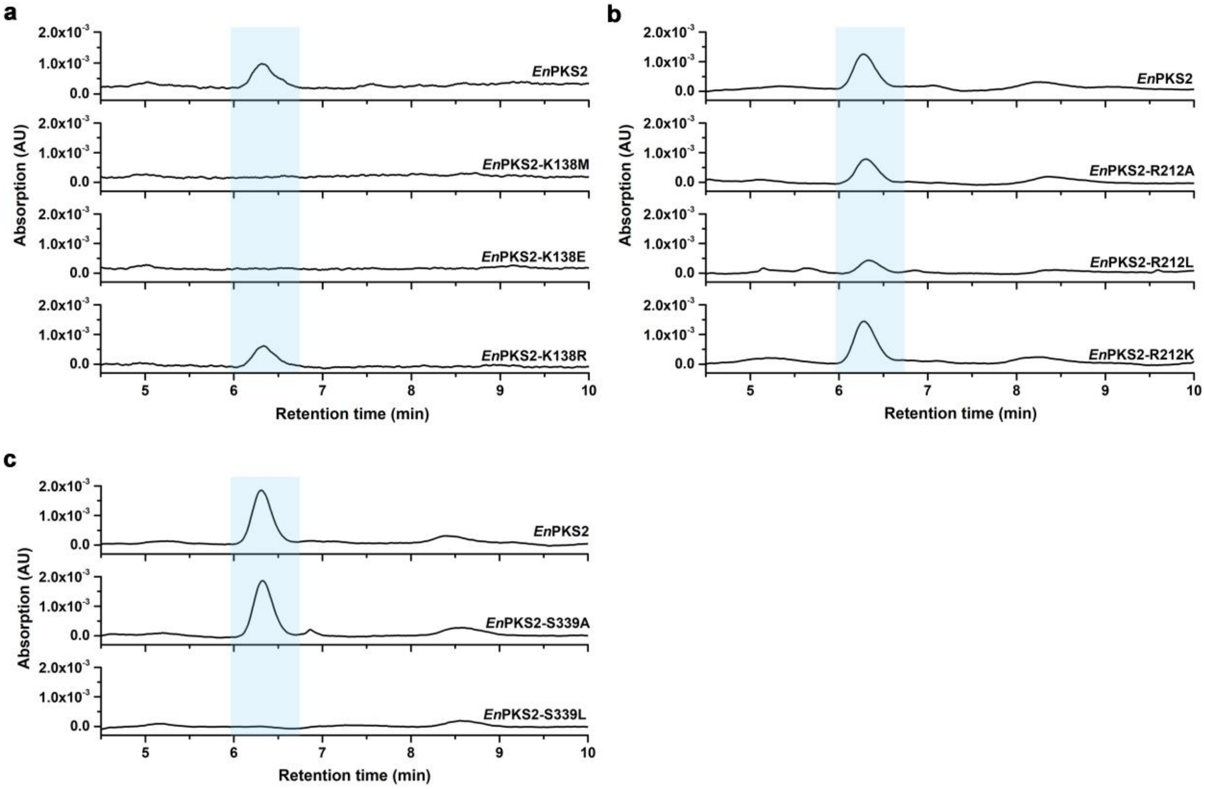

Supplementary Figure 8. HPLC profiles of enzyme products catalyzed by wild type *En*PKS2 and its variants. (**a**) K138M, K138E, and K138R mutants of *En*PKS2. (**b**) R212A, R212L and R212K mutants of *En*PKS2. (**c**) S339L and S339A mutants of *En*PKS2. The products were detected at 242 nm and the UV peaks of enzyme products (OGA, **7**) were marked with cyan shadow. A representative result of n = 3 independent experiments is shown.

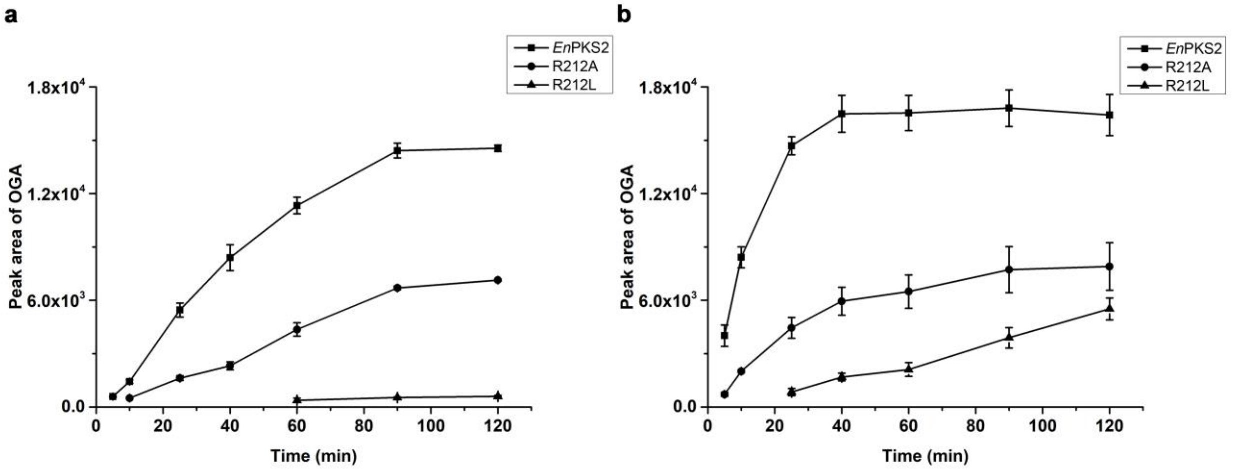

Supplementary Figure 9. Time-course of enzyme reactions catalyzed by wild type *En*PKS2 and its variants. (**a**) The final concentration of malonyl-CoA at 0.5 mM. (**b**) The final concentration of malonyl-CoA at 1.0 mM. The product OGA catalyzed by R212L mutant in the initial period (from 5 min to 40 min for **a**; from 5 min to 10 min for **b** was too low for detection. The productions of OGA catalyzed by wild type *En*PKS2 gradually reached equilibrium after 60 min (for **a**) or 25 min (for **b**). The specific activities of R212A and R212L mutants (61.5%-82.2% decreases for R212A and 94.2%-96.6% decreases for R212L) were counted before the reactions reached equilibrium. Values are the means ± SD of n = 3 independent experiments.

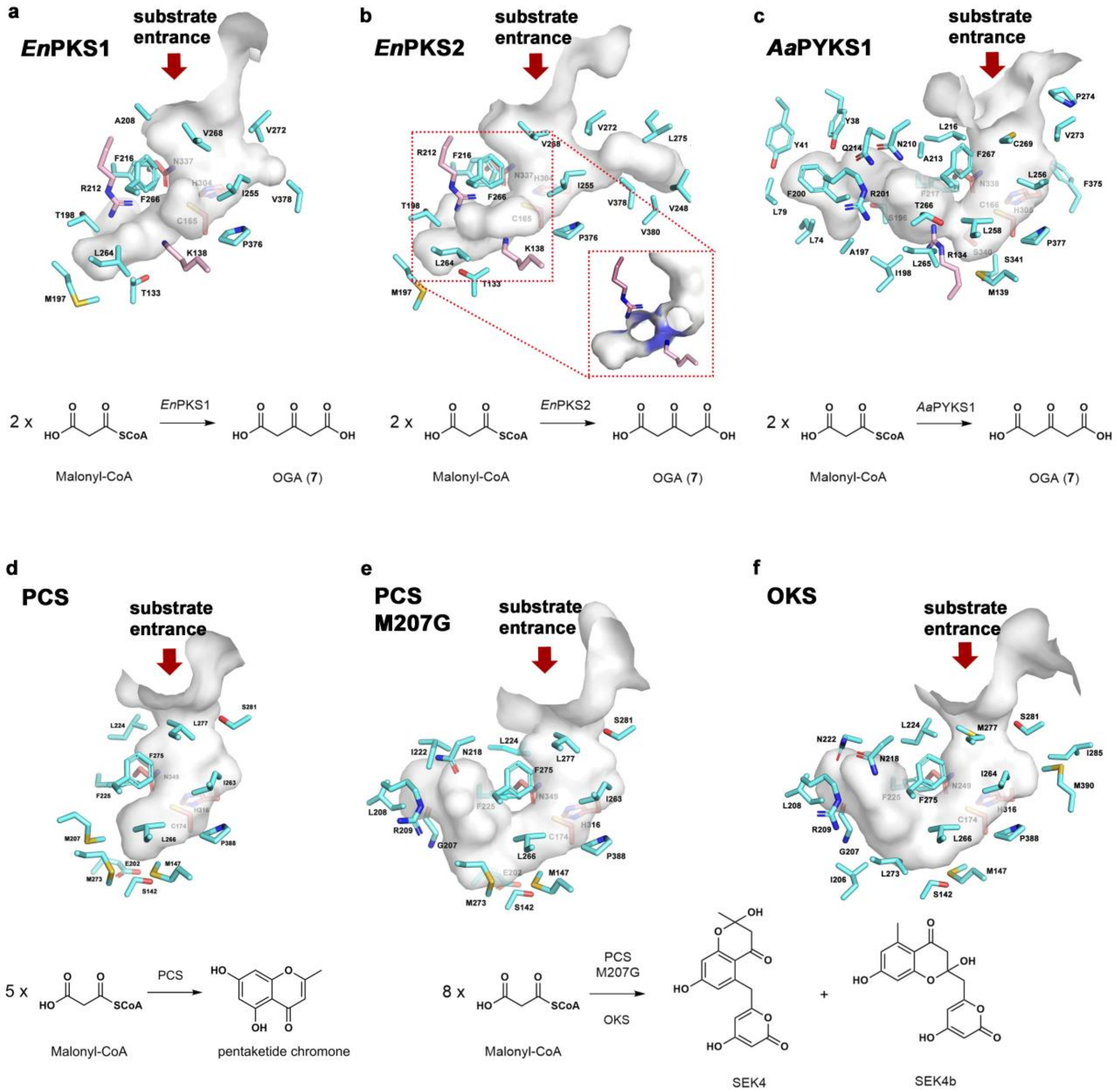

Supplementary Figure 10. Comparison of the active-site architectures of *En*PKS1/2, *Aa*PYKS1, pentaketide chromone synthase (PCS), PCS M207G mutant, and octaketide synthase (OKS). (**a**) The active-site architecture of *En*PKS1 and *En*PKS1-catalyzed reaction. (**b**) The active-site architecture of *En*PKS2 and *En*PKS2-catalyzed reaction. The narrow constriction defined by K138 and R212 in *En*PKS2 is highlighted. (**c**) The active-site architecture of *Aa*PYKS1 and *Aa*PYKS1-catalyzed reaction. (**d**) The active-site architecture of PCS and PCS-catalyzed reaction. (**e**) The active-site architecture of PCS M207G and PCS M207G-catalyzed reaction. (**f**) The active-site architecture of OKS and OKS-catalyzed reaction. The C-H-N catalytic triad are shown as salmon stick models. K138 and R212 in *En*PKS1/2 and R134 in *Aa*PYKS1 are shown as pink stick models.

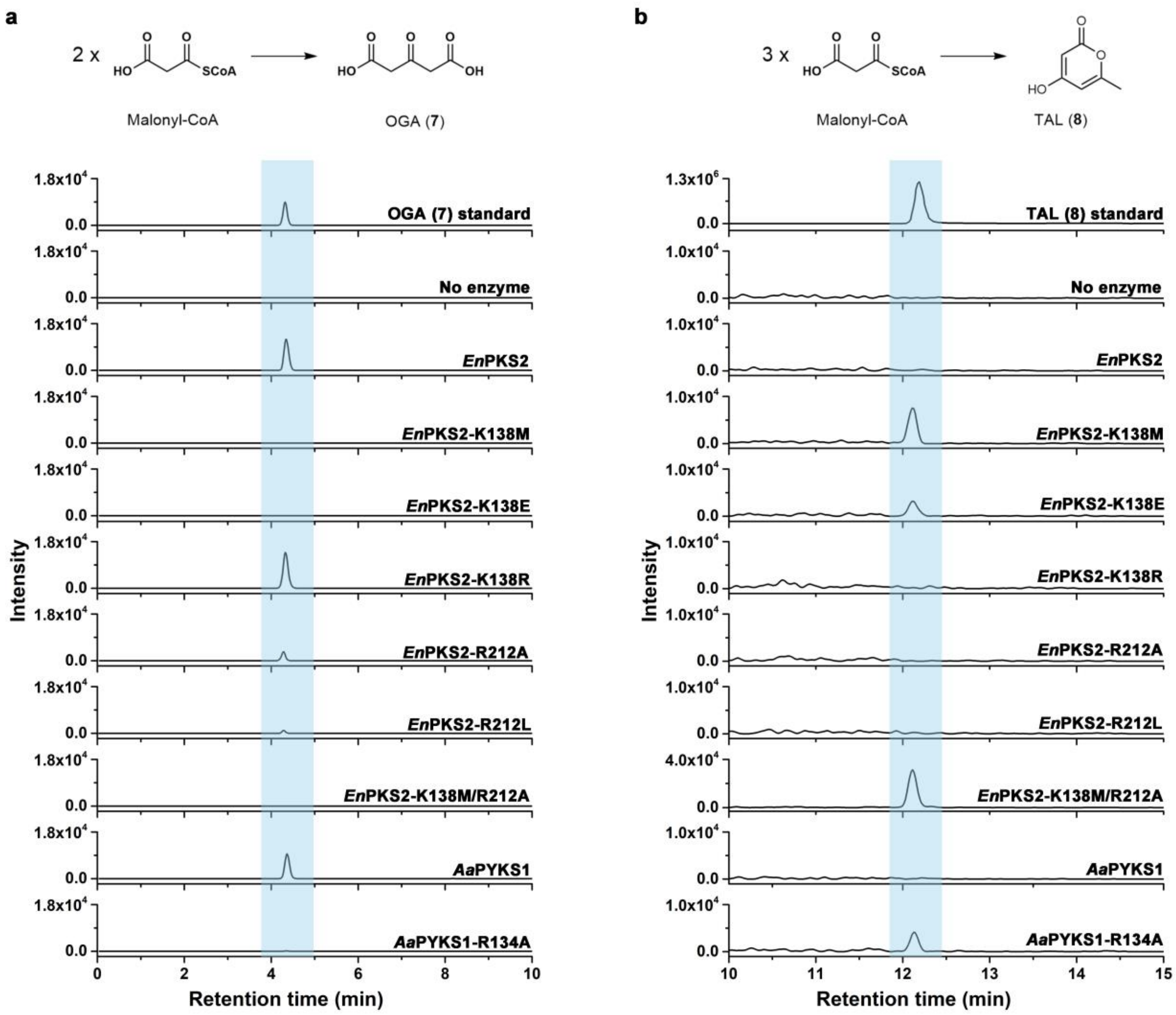

Supplementary Figure 11. Product analysis of the reactions catalyzed by site-directed mutants at K138 and/or R212 of *En*PKS2, or at R134 of *Aa*PYKS1. (**a**) LC-MS chromatograms at $[M + Na]^+$ = 169.0107 ± 0.0010 of OGA (**7**) in enzymatic reactions using malonyl-CoA as substrate. (**b**) LC-MS chromatograms at $[M + H]^+$ = 127.0390 ± 0.0010 of TAL (**8**) in enzymatic reactions using malonyl-CoA as substrate. A representative result of n = 3 independent experiments is shown.

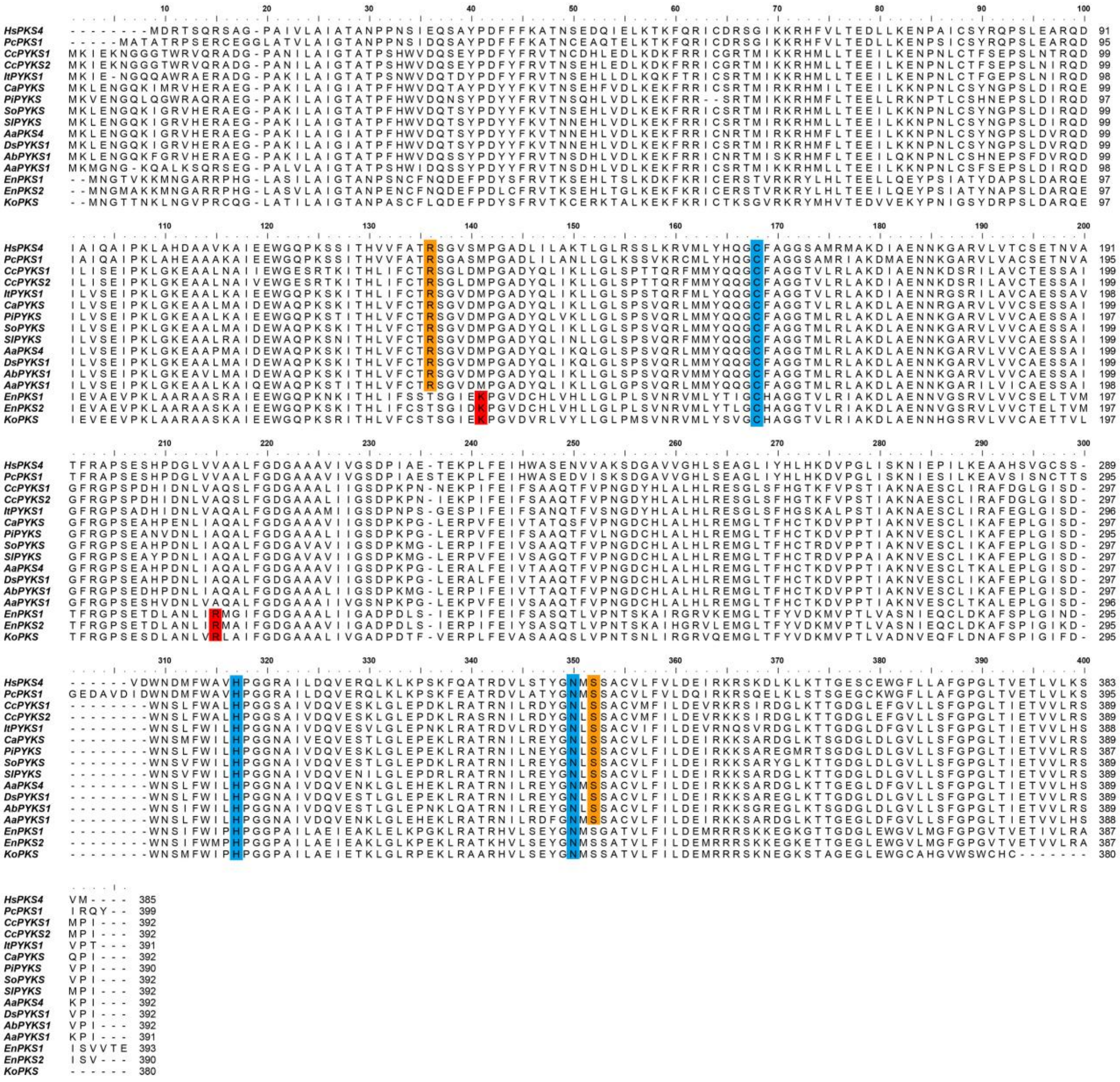

Supplementary Figure 12. Multiple sequence alignment of PKSs. The catalytic triad C-H-N in type III PKS is colored in blue. The arginine (R) and serine (S) residues important for the catalytic activity in PYKS are highlighted in orange. The lysine (K) and arginine (R) residues important for the catalytic activity in *En*PKS1/2 are highlighted in red.

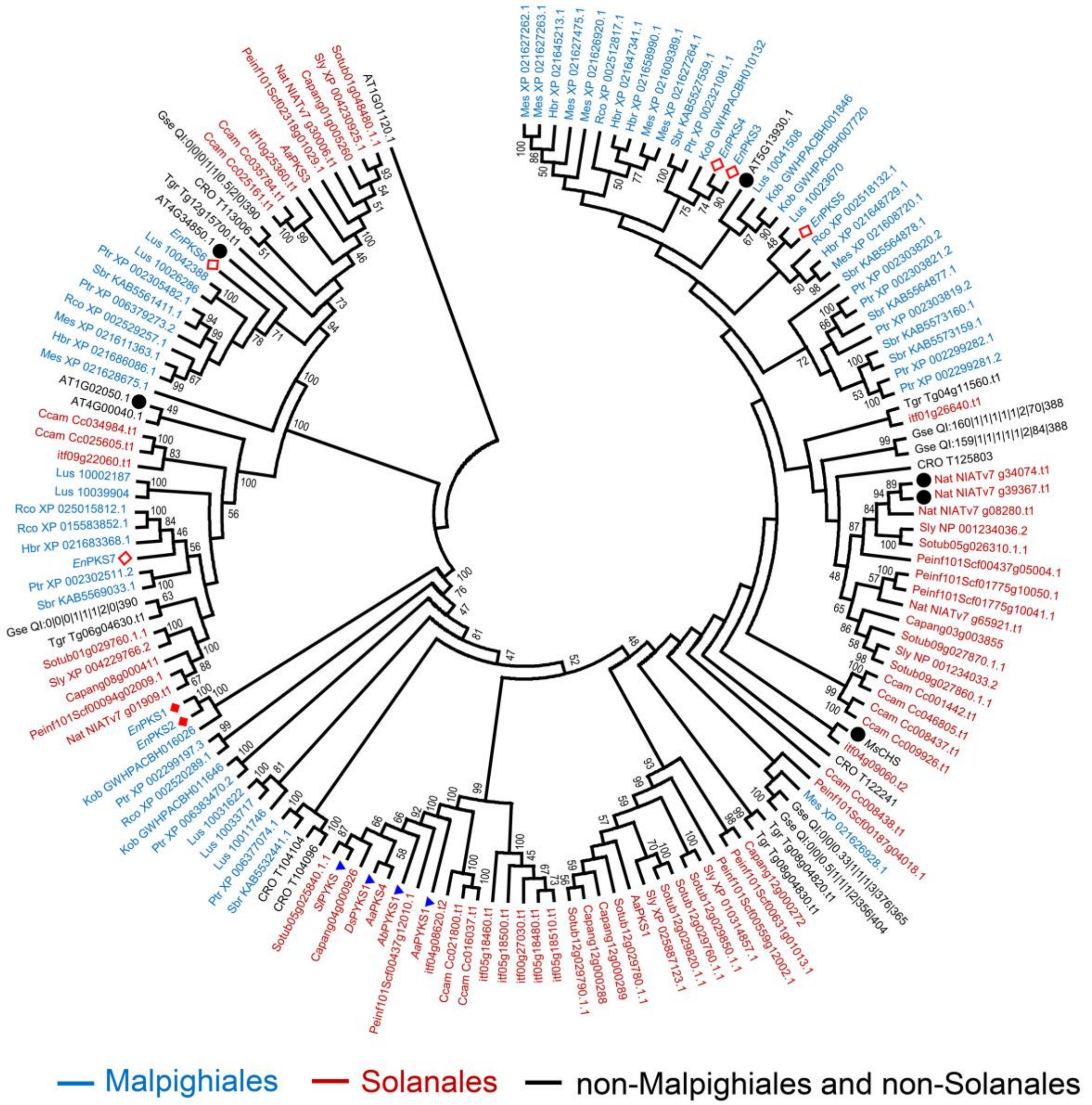

Supplementary Figure 13. The phylogenetic tree of PKSs in Malpighiales and Solanales species. The functionally characterized PKSs are labeled with filled circles, diamonds (*En*PKS1/2) and triangles (*Sl*PYKS, *Ds*PYKS1, *Ab*PYKS1, and *Aa*PYKS1).

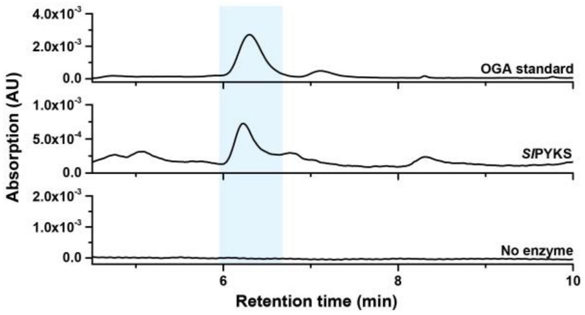

Supplementary Figure 14. HPLC analysis of *Sl*PYKS-catalyzed enzymatic reaction product. The product was detected at 242 nm and the UV peaks of enzyme product (OGA, **7**) were marked with cyan shadow. A representative result of n = 3 independent experiments is shown.

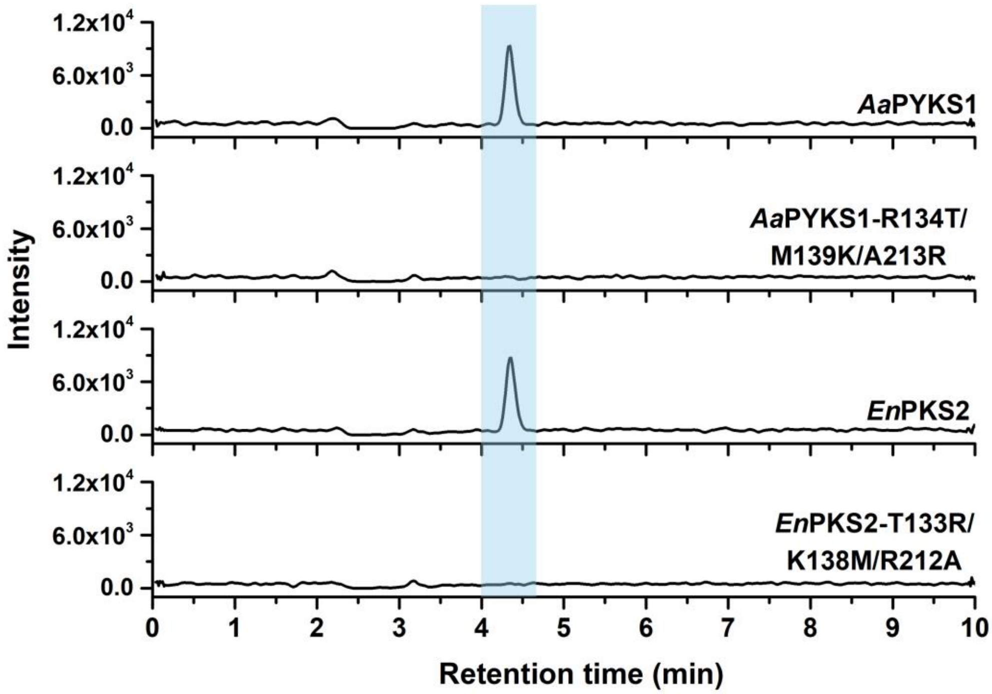

Supplementary Figure 15. OGA-forming activity analysis of R134T/M139K/A213R triple mutant of *Aa*PYKS1 and T133R/K138M/R212A triple mutant of *En*PKS2. A representative result of n = 3 independent experiments is shown.

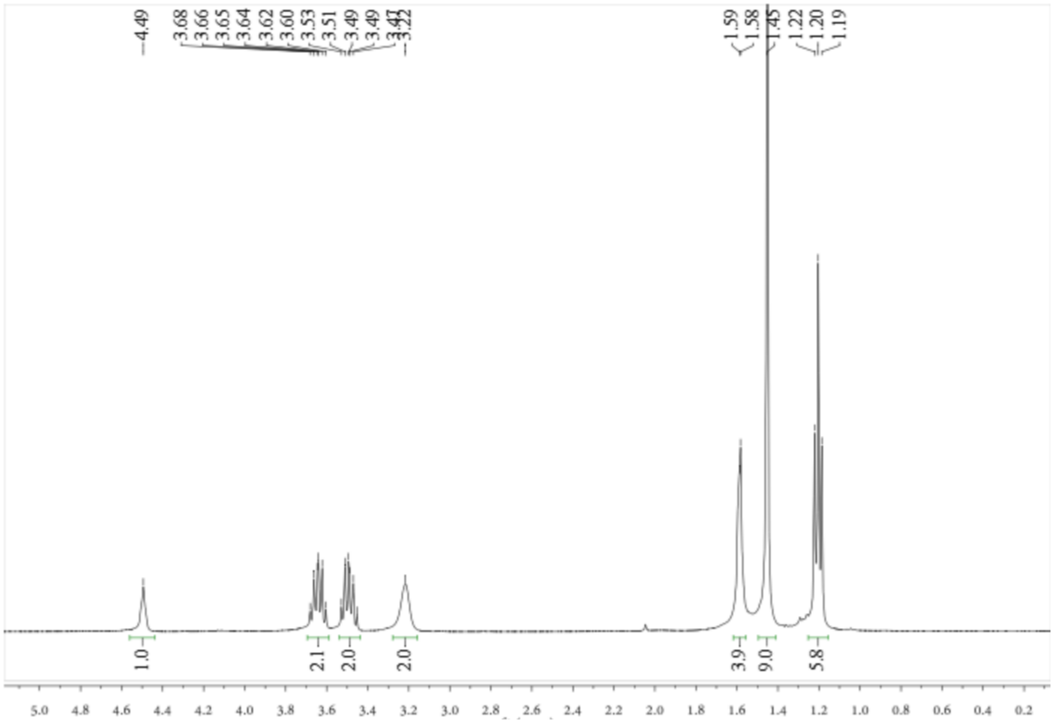

Supplementary Figure 16. The ^{1}H NMR spectrum of s-2 in $CDCl_3$

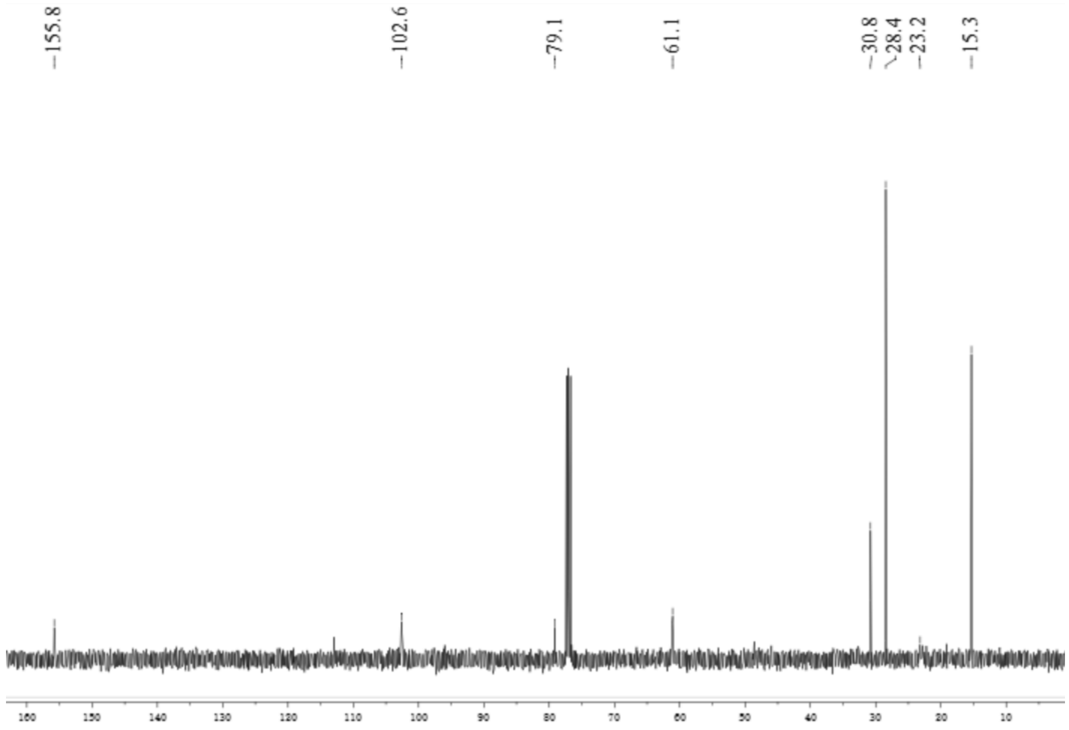

Supplementary Figure 17. The ^{13}C NMR spectrum of s-2 in $CDCl_3$.

User Spectra

Fragmentor Voltage	Collision Energy	Ionization Mode
135	0	ESI

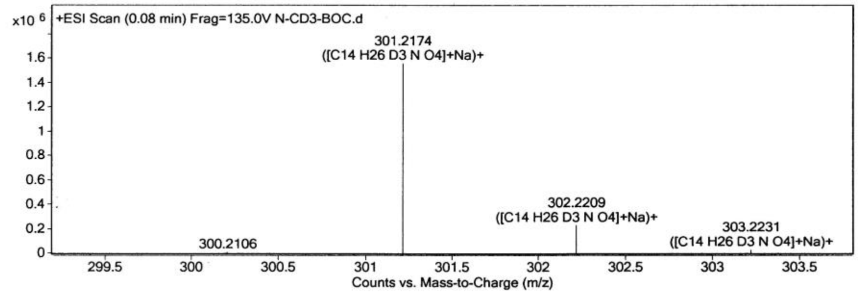

Peak List

m/z	z	Abund	Formula	Ion
134.0805	1	27981.21		
150.1119	1	48186.43		
172.0939	1	125381.8		
233.1932	1	21916.16		
245.1543	1	56005.89		
301.2174	1	1558122.63	C14 H26 D3 N O4	(M+Na)+
302.2209	1	228315.97	C14 H26 D3 N O4	(M+Na)+
303.2231	1	26518.63	C14 H26 D3 N O4	(M+Na)+
317.1913	1	112416.2		
324.2928	1	25192.51		

Formula Calculator Element Limits

Element	Min	Max
C	3	60
H	0	120
O	0	30
D	0	5
N	0	3

Formula Calculator Results

Formula	CalculatedMass	CalculatedMz	Mz	Diff. (mDa)	Diff. (ppm)	DBE
C14 H26 D3 N O4	278.2285	301.2177	301.2174	0.30	1.00	1.0000

--- End Of Report ---

Supplementary Figure 18. The HRMS of s-2.

User Spectra

Fragmentor Voltage	Collision Energy	Ionization Mode
100	0	ESI

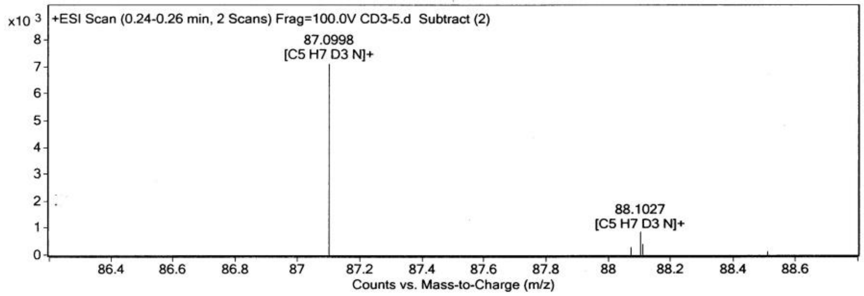

Peak List

m/z	z	Abund	Formula	Ion
87.0998	1	7122.36	C5 H7 D3 N	M+
88.1027	1	837.93	C5 H7 D3 N	M+
105.1099	1	9670.38		
106.1137	1	948.28		
163.1223	1	617.59		
208.0054	1	888.97		
227.1443	1	774.66		
273.1856	1	5305.34		
274.1869	1	707.23		
432.2377	1	656.96		

Formula Calculator Element Limits

Element	Min	Max
C	3	60
H	0	120
O	0	30
N	0	2
D	0	5

Formula Calculator Results

Formula	CalculatedMass	CalculatedMz	Mz	Diff. (mDa)	Diff. (ppm)	DBE
C5 H7 D3 N	87.1002	87.0996	87.0998	-0.20	-2.30	1.5000

--- End Of Report ---

Supplementary Figure 19. The HRMS of [*N*-CD_3] *N*-methyl-Δ^1-pyrrolinium (5).

Supplementary Table 1. Amino acid sequence similarities of *En*PKSs and *Aa*PYKS1.

**Protein**	*En*PKS1	*En*PKS2	*En*PKS3	*En*PKS4	*En*PKS5	*En*PKS6	*En*PKS7	*Aa*PYKS1
*En*PKS1	-	91.79%	62.6%	63.12%	62.44%	40.85%	41.21%	54.55%
*En*PKS2	-	-	65.8%	66.67%	64.91%	40.77%	41.74%	56.36%
*En*PKS3	-	-	-	92.8%	86.12%	41.11%	41.02%	69.66%
*En*PKS4	-	-	-	-	86.38%	41.38%	40.48%	68.16%
*En*PKS5	-	-	-	-	-	41.6%	42.9%	71.13%
*En*PKS6	-	-	-	-	-	-	67.56%	39.62%
*En*PKS7	-	-	-	-	-	-	-	40.59%
*Aa*PYKS1	-	-	-	-	-	-	-	-

Supplementary Table 2. Kinetic parameters of *En*PKS1/2 and *Aa*PYKS1.

	***K*m (μM)**	***K*cat (s^{-1})**	***K*cat/*K*m (s^{-1} M^{-1})**
*En*PKS1	112.90	1.89	16768.88
*En*PKS2	50.54	0.45	8975.48
*Aa*PYKS1	70.38	2.24	31860.63

Supplementary Table 3. Data collection and refinement statistic.

	***En*PKS1(PDB ID: 7F0G)**	***En*PKS2(PDB ID: 7F0E)**
**Data collection**		
Space group	*P*1	$P2_12_12_1$
Cell dimensions		
a, b, c (Å)	52.08, 117.86, 136.47	95.04, 112.82, 154.48
α β γ (°)	111.34, 90.62, 91.59	90.00, 90.00, 90.00
Resolution (Å)	66.49- 2.67 (2.74- 2.67)	50.00 -2.62 (2.69-2.62)
Wavelength (Å)	0.979	0.979
R_{merge}	0.096(0.362)	0.173(1.370)
R_{pim}	0.061(0.267)	0.052(0.421)
$I/\sigma I$	10.9(3.3)	11.5(2.1)
Completeness (%)	94.3(90.1)	99.7(99.6)
Redundancy	3.4(2.7)	13.1(12.8)
**Refinement**		
Resolution (Å)	23.07- 2.67	91.22-2.62
No. reflections	80450	50653
R_{work}/R_{free}	0.197/0.221	0.196/0.248
No. atoms		
Protein	23761	1500
Water	435	65
B-factors		
Protein	32.4	58.3
Water	31.2	53.1
R.m.s. deviations		
Bond lengths (Å)	0.013	0.012
Bond angles (°)	1.798	1.922

Supplementary Table 4. The primers used in this study.

Primer names	Primers	Target plasmid
*En*PKS1-28a-*Nde*I-F	tggtgccgcgcggcagccatATGAACGGAACCGTCAAGAA	pET28a-*En*PKS1
*En*PKS1-28a-*Bam*HI-R	cggagctcgaattcggatccTCATTCTGTAACCACAGAAA	
*En*PKS2-28a-*Nde*I-F	tggtgccgcgcggcagccatATGAACGGAATGGCTAAGAA	pET28a-*En*PKS2
*En*PKS2-28a-*Bam*HI-R	cggagctcgaattcggatccTCACACAGAAATGGCACGCA	
*En*PKS2-pCold-*Bam*HI-F	tggagctcggtacccTCGAGATGAACGGAATGGCTAAGAA	pCold-*En*PKS2
*En*PKS2-pCold-*Sal*I-R	acaagcttgaattcggatccTCACACAGAAATGGCACGCA	
*En*PKS2-pCold-R212A-F	TTAGCTAATCTTATAGCTATGGCAATTT	pCold-*En*PKS2-R212A; pCold-*En*PKS2-K138M/R212A; pCold-*En*PKS2-T133R/K138M/R212A
*En*PKS2-pCold-R212A-R	GCTATAAGATTAGCTAAGTCAGTCTCC	
*En*PKS2-pCold-R212L-F	TTAGCTAATCTTATACTTATGGCAATTT	pCold-*En*PKS2-R212L
*En*PKS2-pCold-R212L-R	AGTATAAGATTAGCTAAGTCAGTCTCC	
*En*PKS2-pCold-R212K-F	TTAGCTAATCTTATAAAGATGGCAATTT	pCold-*En*PKS2-R212K
*En*PKS2-pCold-R212K-R	CTTTATAAGATTAGCTAAGTCAGTCTCC	
*En*PKS2-pCold-K138M-F	TTCTGGGATAGACATGCCTGGCGTTG	pCold-*En*PKS2-K138M; pCold-*En*PKS2-K138M/R212A; pCold-*En*PKS2-T133R/K138M/R212A
*En*PKS2-pCold-K138M-R	ATGTCTATCCCAGAAGTTGAACAGAAG	
*En*PKS2-pCold-S339A-F	GAATATGGGAACATGGCAAGCGCGAC	pCold-*En*PKS2-S339A
*En*PKS2-pCold-S339A-R	CCATGTTCCCATATTCACTCAGGACA	
*En*PKS2-pCold-S339L-F	GAATATGGGAACATGTTAAGCGCGAC	pCold-*En*PKS2-S339L
*En*PKS2-pCold-S339L-R	AACATGTTCCCATATTCACTCAGGACA	
*En*PKS2-pCold-K138E-F	TTCTGGGATAGACGAGCCTGGCGTTG	pCold-*En*PKS2-K138E
*En*PKS2-pCold-K138E-R	CGTCTATCCCAGAAGTTGAACAGAAG	
*En*PKS2-pCold-K138R-F	TTCTGGGATAGACAGGCCTGGCGTTG	pCold-*En*PKS2-K138R
*En*PKS2-pCold-K138R-R	CTGTCTATCCCAGAAGTTGAACAGAAG	
*En*PKS2-pCold-T133R-F	CTCATCTTCTGTTCACGTTCTGGGATAG	pCold-*En*PKS2-T133R/K138M/R212A
*En*PKS2-pCold-T133R-R	CGTGAACAGAAGATGAGATGGGTGATC	
AaPYKS1-pCold-F	tcggtaccctcgagggatccATGAAGATGGGAAATGGTAA	pCold-*Aa*PYKS1
AaPYKS1-pCold-R	tatctagactgcaggtcgacTTAAATGGGCTTACTATGGA	
AaPYKS1-pCold-R134A-F	GTCTTTTGCACTGCAAGCGGCGTAG	pCold-*Aa*PYKS1-R134A
AaPYKS1-pCold-R134A-R	GCAGTGCAAAAGACTAAATGGG	
AaPYKS1-pCold-R134T/M139K-F	ACAAGCGGCGTAGACAAGCCCGGGGCCGATTA	pCold-*Aa*PYKS1-R134T/M139K/A213R
AaPYKS1-pCold-R134T/M139K-R	CTTGTCTACGCCGCTTGTAGTGCAAAAG	
AaPYKS1-pCold-A213R-F	GCGCCAAGCATTGTTTGG	pCold-*Aa*PYKS1-R134T/M139K/A213R
AaPYKS1-pCold-A213R-R	GCTTGGCGCACAAGATTATCAAC	
*Aa*PYKS-pET28a-F	agcaaatgggtcgcggatccATGAAGATGGGAAATGGTAA	pET28a-*Aa*PYKS
*Aa*PYKS-pET28a-R	gcggccgcaagcttgtcgacTTAAATGGGCTTACTATGGA	
*Sl*PYKS-pET28a-F	GGATCCATGAAACTGGAAAACGGTC	pET28a-*Sl*PYKS
*Sl*PYKS-pET28a-R	GTCGACTTAAATCGGCATGCTG	
*En*PKS3-28a-*Nde*I-F	tggtgccgcgcggcagccatATGGCCACCGTTGACGATAT	pET28a-*En*PKS3
*En*PKS3-28a-*Bam*HI-R	cggagctcgaattcggatccTCAATGAGTGGGTACGCTAT	
*En*PKS4-28a-*Nde*I-F	tggtgccgcgcggcagccatATGGTCACCCTTGAAGAGAT	pET28a-*En*PKS4
*En*PKS4-28a-*Bam*HI-R	cggagctcgaattcggatccTCAGTGACCGGTGGGAACGC	
*En*PKS5-28a-*Nde*I-F	tggtgccgcgcggcagccatATGGTGAGCGTTGATGAAGT	pET28a-*En*PKS5
*En*PKS5-28a-*Bam*HI-R	cggagctcgaattcggatccTTAAGTGGCAATGCTATGGA	
*En*PKS6-28a-*Nde*I-F	tggtgccgcgcggcagccatATGGGCAGCGAAGAGACTGT	pET28a-*En*PKS6
*En*PKS6-28a-*Bam*HI-R	cggagctcgaattcggatccTCAGAGAGTGAGGTTCCTTG	
*En*PKS7-28a-*Nde*I-F	tggtgccgcgcggcagccatATGGCAAGAACTGACAGCAA	pET28a-*En*PKS7
*En*PKS7-28a-*Bam*HI-R	cggagctcgaattcggatccTTACAGGCTTCGAAGAAGGA	

Supplementary References

1. Huang, J.-P., *et al.* Tropane alkaloids biosynthesis involves an unusual type III polyketide synthase and non-enzymatic condensation. *Nat. Commun.* **10**, 4036 (2019).
2. Jiang, C., Kim, S. Y. & Suh, D. Y. Divergent evolution of the thiolase superfamily and chalcone synthase family. *Mol. Phylogenet. Evol.* **49**, 691-701 (2008).
