# Supplementary material for: Catalytic innovation underlies independent recruitment of polyketide synthases in cocaine and hyoscyamine biosynthesis
Source: Nat Commun. 2022 Aug 25;13:4994. doi: 10.1038/s41467-022-32776-1 (PMC9411544; doi:10.1038/s41467-022-32776-1)
Supplement: Supplementary file 2 — Reporting Summary [file 41467_2022_32776_MOESM2_ESM.pdf]

nature portfolio

Corresponding author(s): Sheng-Xiong Huang

Last updated by author(s): Aug 3, 2022

Reporting Summary

Nature Portfolio wishes to improve the reproducibility of the work that we publish. This form provides structure for consistency and transparency in reporting. For further information on Nature Portfolio policies, see our Editorial Policies and the Editorial Policy Checklist.

Statistics

For all statistical analyses, confirm that the following items are present in the figure legend, table legend, main text, or Methods section.

n/a	Confirmed	
☐	☒	The exact sample size (*n*) for each experimental group/condition, given as a discrete number and unit of measurement
☐	☒	A statement on whether measurements were taken from distinct samples or whether the same sample was measured repeatedly
☒	☐	The statistical test(s) used AND whether they are one- or two-sided<br>*Only common tests should be described solely by name; describe more complex techniques in the Methods section.*
☒	☐	A description of all covariates tested
☒	☐	A description of any assumptions or corrections, such as tests of normality and adjustment for multiple comparisons
☐	☒	A full description of the statistical parameters including central tendency (e.g. means) or other basic estimates (e.g. regression coefficient) AND variation (e.g. standard deviation) or associated estimates of uncertainty (e.g. confidence intervals)
☒	☐	For null hypothesis testing, the test statistic (e.g. *F*, *t*, *r*) with confidence intervals, effect sizes, degrees of freedom and *P* value noted<br>*Give P values as exact values whenever suitable.*
☒	☐	For Bayesian analysis, information on the choice of priors and Markov chain Monte Carlo settings
☒	☐	For hierarchical and complex designs, identification of the appropriate level for tests and full reporting of outcomes
☒	☐	Estimates of effect sizes (e.g. Cohen's *d*, Pearson's *r*), indicating how they were calculated

Our web collection on statistics for biologists contains articles on many of the points above.

Software and code

Policy information about availability of computer code

Data collection	Chromaster was used for HPLC data collection. Agilent MassHunter were used for enzymatic data collection. MEGA (version 6) was used for amino acid alignment and phylogenetic tree construction. ChemBioDraw Ultra 14.0 was used for drawing chemical structures. The diffraction data of protein crystals were processed and scaled with XDS (BUILT=20210205). Initial model was build using Phenix 1.0. Manual adjustment of the model was carried out using the program Coot-0.9.4 and the models were refined by Phenix 1.0 and Refmac5.
Data analysis	OriginPro 9.0 was used for LC-MS and HPLC data visualization. GraphPad Prism 7 was used for Michaelis-Menten kinetic analysis. Coot-0.9.4 and Phenix 1.0 were used to perform protein structure model building. PyMOL 2.4 was used for protein structure visualization.

For manuscripts utilizing custom algorithms or software that are central to the research but not yet described in published literature, software must be made available to editors and reviewers. We strongly encourage code deposition in a community repository (e.g. GitHub). See the Nature Portfolio guidelines for submitting code & software for further information.

Data

Policy information about availability of data

All manuscripts must include a data availability statement. This statement should provide the following information, where applicable:

- Accession codes, unique identifiers, or web links for publicly available datasets
- A description of any restrictions on data availability
- For clinical datasets or third party data, please ensure that the statement adheres to our policy

The transcriptome datasets of E. novogranatense have been deposited in NCBI under accession numbers SRR15399168 to SRR15399180. The gene sequences of

EnPKS1 and EnPKS2 have been deposited in GenBank under accession numbers MZ819697 and MZ819696. The atomic models of EnPKS1 and EnPKS2 have been deposited in the Protein Data Bank under PDB IDs 7F0G and 7F0E. All data that support the findings of this study are available in the main text and the supplementary information. Source data are provided with this paper.

Human research participants

Policy information about studies involving human research participants and Sex and Gender in Research.

Reporting on sex and gender	It is not relevant to this study.
Population characteristics	It is not relevant to this study.
Recruitment	It is not relevant to this study.
Ethics oversight	It is not relevant to this study.

Note that full information on the approval of the study protocol must also be provided in the manuscript.

Field-specific reporting

Please select the one below that is the best fit for your research. If you are not sure, read the appropriate sections before making your selection.

- [x] Life sciences
- [ ] Behavioural & social sciences
- [ ] Ecological, evolutionary & environmental sciences

For a reference copy of the document with all sections, see nature.com/documents/nr-reporting-summary-flat.pdf

Life sciences study design

All studies must disclose on these points even when the disclosure is negative.

Sample size	No sample size calculation was performed. Replicates of n≥2 were chosen for in vitro and in vivo enzyme activity assays, as they are sufficient for us to assess the presence/absence of a peak or mass signature (representative of a metabolite).
Data exclusions	No data was excluded.
Replication	All experiments describing the function of wild type enzymes or their variants were performed at least 3 times, with similar results consistently observed. X-ray data collected were from single crystals. Representative SDS-PAGE gel image from three independent experiments was shown. All attempts at replication were successful.
Randomization	For heterologous expression in tobacco leaves, the replicates were randomized among different plants.
Blinding	Complete blinding was not feasible for this study, as samples were prepared and measured by the same researcher. Besides, these experiments required the insight of the researcher into the enzymes/genes and/or metabolites being analyzed.

Reporting for specific materials, systems and methods

We require information from authors about some types of materials, experimental systems and methods used in many studies. Here, indicate whether each material, system or method listed is relevant to your study. If you are not sure if a list item applies to your research, read the appropriate section before selecting a response.

Materials & experimental systems

n/a	Involved in the study	
x		Antibodies
x		Eukaryotic cell lines
x		Palaeontology and archaeology
x		Animals and other organisms
x		Clinical data
x		Dual use research of concern

Methods

n/a	Involved in the study	
x		ChIP-seq
x		Flow cytometry
x		MRI-based neuroimaging
